# Associations of dietary and drinking water habits with number of natural teeth: a longitudinal study in the Chinese elderly population

**DOI:** 10.1186/s12877-021-02473-7

**Published:** 2021-10-02

**Authors:** Dan Zhao, Jia Ning, Yifei Zhao, Eryi Lu

**Affiliations:** 1 Department of Stomatology, Renji Hospital, School of Medicine, Shanghai Jiao Tong University, 200127 Shanghai, China; 2 School of Stomatology, Tianjin Medical University, No 22. Qixiangtai Road, Heping District, 300070 Tianjin, China

**Keywords:** Dietary intake, Water, Oral health, Teeth loss, Cohort study

## Abstract

**Background:**

The relationship between dietary and drinking water habits and oral health are still unclear. We aimed at evaluating the association of dietary and drinking water habits with number of teeth in the elderly adults.

**Methods:**

We conducted a longitudinal study based on the Chinese Longitudinal Healthy Longevity Survey from 1998 to 2018. The data of dietary and drinking water habits at baseline were collected using a questionnaire. The number of teeth at baseline and follow-up was collected for each subject. We used the linear mixed-effect model to analyze the associations of dietary habits and drinking water sources with tooth number.

**Results:**

Among 19,896 participants at baseline, the mean age of the participants was 83.87 years, with the average number of natural teeth of 9.37, 8.26, 8.38, 8.68, 4.05, 1.92, 1.12, 2.20 for the first to eighth waves of survey. Compared with subjects drinking tap water, 1.036 (*95 % CI*: -1.206, -0.865), 0.880 (*95 % CI*: -1.122, -0.637) and 1.331 (*95 % CI*: -1.715, -0.947) fewer natural teeth were reported for those drinking well, surface water and spring at baseline survey. Compared with participants with rice intake as the staple food, those with wheat intake (*β* = -0.684; *95 % CI*: -0.865, -0.503) tended to have fewer natural teeth. Compared with participants with fresh fruit intake almost every day, those with quite often intake of fresh fruit tended to have fewer teeth with a significant dose-response trend (*P*_*trend*_ <0.001). Similar decreased trend for number of teeth was also indicated for increased frequency of vegetable intake (*P*_*trend*_ <0.001). Fewer number of teeth was found for subjects with less frequency of meat and fish intakes.

**Conclusions:**

The study suggested that drinking well, surface water, and spring, intakes of wheat as staple food, as well as less frequency of fresh fruit, vegetable, meat and fish intakes were associated with significantly fewer number of teeth in the Chinese elderly population.

**Supplementary Information:**

The online version contains supplementary material available at 10.1186/s12877-021-02473-7.

## Background

Tooth loss is a major problem of oral health in older adults. An increase in life expectancy results in a decrease in the number of teeth. [1] It is reported that nearly 20 % adults aged 65 or older have lost all of their teeth. Complete tooth loss is twice as prevalent among adults aged 75 and older (26 %) compared with adults aged 65–74 (13 %). [2] Tooth loss and its underlying mechanisms have been broadly associated with some systemic diseases such as cardiovascular disease, diabetes and cancer, which lead to huge social and economic burden. [3, 4] Therefore, it is significant to find out the influencing factors of tooth loss.

Tooth loss or wearing dentures can affect nutrition because people without teeth or with dentures often prefer soft, easily chewed foods instead of foods such as fresh fruits and vegetables. [5] Several large cross-sectional studies have reported that impaired dentition status is associated with poor dietary intake. [6] The intakes of vegetable, fish, shellfish and their products were significantly lower among subjects with fewer teeth. [6] Besides correlation with the foods, large cross-sectional study in elderly Japanese also suggested that the number of teeth present had a significant relationship with the intake of several nutrients, in particular, total protein, animal protein, sodium, vitamin D, vitamin B1, vitamin B6, niacin, and pantothenic acid. [7] Furthermore, previous studies indicated that drinking different waters, such as tap water rich in fluoride, may also affect oral health by the prevention of caries. [8–10] Globally, 355 million people are receiving water fluoridation. [10] However, there are wide variations of fluoride levels in drinking water in China. Guangdong Province is the only province that has experienced artificial water fluoridation in China. [10] During 1965–1983, the fluoridation project was started in Guangzhou, and the average fluoride levels increased from 0.2 to 0.3 to 0.8-1.0 ppm. [10].

 Oral health status has a profound impact on the acquisition and utilization of nutrients; and interchangeably the nutrients intakes may also determine the state of oral health by preventing tooth loss and oral diseases. [11] Previous cross-sectional studies fail to address the temporal relation between dietary intakes and teeth loss. It is hypothesized that the consequence of choice of food and waters in a time period may play a role in an individual’s teeth loss. Herein, we conducted a longitudinal study to evaluate the relationship of dietary intakes and drinking water sources with number of teeth in the Chinese elderly population.

## Methods

### Study population

The Chinese Longitudinal Healthy Longevity Survey (CLHLS) is an ongoing prospective cohort study of Chinese old adults. The CLHLS applied a multistage, stratified cluster sampling design to recruit participants from 22 of the 31 provinces in China. Six hundred and thirty-one counties or cities were randomly selected, representing 85 % of the total Chinese population. The investigation started in 1998, and seven rounds of follow-up surveys were conducted in 2000, 2002, 2005, 2008–2009, 2011–2012, 2014, and 2017–2018. The response rate in each wave of survey is approximately 90 %. To maintain enough sample size, the CLHLS replaced the deceased participants with new participants in the follow-up waves. The questionnaire used in the CLHLS was translated from the instruments of the Danish longevity survey [12] and has been used in previous studies [13–15] (**Supplementary document**). The questionnaire data provide information on various determinants of health, including demographic and socioeconomic characteristics, lifestyle, the capacity of physical performance, cognitive functioning, chronic disease prevalence, social activities, diet, smoking and drinking behaviors, and oral health. We included all participants from the 1998 survey. For the follow-up surveys, the participants included the survivals from the previous waves and the new participants. A total of 57,257 participants in the CLHLS were included in the study. The study was approved by the Institutional Review Board, Duke University (Pro00062871), and the Biomedical Ethics Committee, Peking University (IRB00001052–13,074) in accordance with the principles of the Declaration of Helsinki. And written informed consents were obtained during the face to-face interview. More details of the CLHLS could be obtained elsewhere. [16, 17]

We excluded ineligible participants from the study because of (1) missing information or abnormal values on covariates, such as weight, smoking, drinking, and history of hypertension, diabetes, and cardiovascular disease and so on (n = 11,116); (2) missing information on exposure and outcome (n = 26,096); (3) baseline age less than 60 years (n = 149). Finally, 19,896 participants were included in the final analysis.

### Exposure assessments

Self-reported information on food consumption was collected through face-to-face interviews by trained research staff. The participants were asked to report their food frequency intake of fruit, vegetable, meat, and fish in last month. The frequency of meat and fish was recorded as “almost every day” or “occasionally” or “rarely or never”. If participants consumed meat and fish at least 4 days within a week, the frequency of meat and fish intake was identified as “almost every day”. If they consumed meat and fish 2–3 days within a week, the frequency of meat and fish intake was identified as “occasionally”. If they consumed meat and fish ≤ 1 day within a week, the frequency of meat and fish intake was identified as “rarely or never”. The frequency of fruit and vegetable intake was recorded as “almost every day”, “quite often”, “occasionally” or “rarely or never”. If they consumed fruit and vegetable at least 6 days within a week, the frequency of fruit and vegetable intake was identified as “almost every day”. If they consumed fruit and vegetable 4–5 days within a week, the frequency of fruit and vegetable intake was identified as “quite often”. If they consumed fruit and vegetable 2–3 days within a week, the frequency of fruit and vegetable intake was identified as “occasionally”. If they consumed fruit and vegetable ≤ 1 day within a week, the frequency of fruit and vegetable intake was identified as “rarely or never”.

Staple food pattern was assessed by the question “Please tell us the staple food you eat last month: (1) Rice, (2) Corn (maize), (3) Wheat (noodles and bread, etc.), (4) Half rice and half wheat”. Participants were also asked for the amount of staple food intake in liang per day (liang is a Chinese unit, equals to 50 g).

A question “Water you drank was mainly from at around age 60 years?” was used to identify the drinking water sources. Five sources of drinking water including wells, rivers or lakes, spring, pond or pool, and tap water were reclassified as follows: tap water, wells, surface water, and spring in the final analysis. Similarly, each participant was asked that what kind of water you usually drink to identify drinking habits, including boiled and un-boiled water.

### Outcome assessment

Self-reported information on natural and false teeth, as well as the use of dentures, were collected with the following questions: (1) How many natural teeth do you still have? (2) Do you have false teeth? False teeth included both fixed and removable denture in this study. Data were repeatedly collected and updated in each follow-up wave. The interviewers could help older adults to confirm their responses to these questions, which may ensure the validity of self-reported information on natural teeth and denture use.

### Covariates measurement

Age, sex (male/female), education, living areas, and ethnicity were measured at baseline, and all other covariates (weight, marital status, current smoker, current alcohol consumer, physical activity, self-reported health, history of hypertension, history of diabetes, and history of cardiovascular diseases) were measured at baseline and at each follow-up interview. For example, cigarette smoking status was categorized into non-smokers (representing never smokers) and current smokers. The participants were asked whether they drink alcohol (yes/no) as well as the type and amount of alcohol they consumed. Information on regular physical activity was collected using question “Do you do exercise regularly at present, including jogging, playing ball, running and Qigong?” and recoded as yes or no. Self-rated health was measured via a question: how do you rate your health in general? In the final analysis, five response options including very good, good, fair, poor, and very poor were reclassified as three options: good, fair, and poor. Chronic diseases including hypertension, diabetes and cardiovascular disease were obtained by asking respondents if a physician had ever told them that they had the condition. History of hypertension, diabetes, and cardiovascular diseases was identified using a similar question: Do you suffer from hypertension/ diabetes/cardiovascular diseases?

### Statistical analysis

Descriptive statistics were used to summarize baseline socio-demographic status, medical conditions, and health behaviors. To evaluate the association between dietary factors and number of teeth, a linear mixed-effect model was used to analyze the associations of dietary habits and drinking water sources with tooth number with adjustment for potential confounders. Confounders were identified based on prior knowledge and listed as follows: age, sex, weight, marital status, education, ethnicity, current smoker, current alcohol consumer, physical activity, living areas, false teeth, self-reported health, history of hypertension, history of diabetes, history of cardiovascular diseases, and wave. Subgroup analyses were also conducted based on sex and living areas. Three sensitivity analyses were conducted. In the first sensitivity analysis, in addition to the identified confounders above, we also mutually adjusted drinking habits, drinking water sources at around 60 years, staple food, and frequency of intake fresh fruit, vegetables, meat, and fish in the model. In the second sensitivity analysis, the associations of drinking water and dietary habits with number of natural teeth were analyzed with excluding participants with physical and cognitive impairment. In the third sensitivity analysis, to analyze the influence of missing data, multiple imputation was employed to impute missing data in all variables. And the complete dataset was used to analyze the associations of drinking water and dietary habits with number of natural teeth. All analyses were conducted using SAS 9.4 (SAS Institute, Inc., Cary, NC, USA). *P* < 0.05 was considered statistically significant.

## Results

### Baseline characteristics

Table 1 shows the sample characteristics at baseline. The mean age of the participants was 83.87 (standard deviation: 11.14) years. The drinking water sources at round 60 years were from well water (56.03 %), tap water (26.95 %), surface water (13.25 %) and spring (3.76 %). About 70.01 % of the participants lived in urban areas. Moreover, 65.03 % of the participants had rice as their staple food, 64.52 % of the participants had vegetables almost every day, and more than 68 % of participants had fruit occasionally or rarely. For the frequency of meat intake, 45.97 % and 37.99 % of the participants ate meat almost every day or occasionally, and 16.03 % rarely or never ate meat. About 29.98 % of the participants ate fish almost every day. The proportions of current alcohol drinking and tobacco smoking were 22.61 % and 21.13 %, respectively. For major chronic diseases history, 59.02 % of the participants reported history of hypertension, 4.09 % had cardiovascular disease history, and 1.85 % had diabetes. The average number of natural teeth were 9.37, 8.26, 8.38, 8.68, 4.05, 1.92, 1.12, 2.20 for the first to eighth waves of survey. About 27.87 % of the participants had false teeth at baseline.

**Table 1:** The characteristics at baseline of all participants (*N* = 19,896)

Characteristics	Mean ± Standard deviation	Number of participants (%)
Age (years)	83.87 ± 11.14	-
Weight (kg)	50.42 ± 11.15	-
Sex		-
Male	-	8,984(45.15)
Female	-	10,912(54.85)
Marital status		
Married	-	7,628(38.34)
Divorced	-	95(0.48)
Widowed	-	11,955(60.09)
Unmarried	-	218(1.10)
Living areas		
Urban	-	13,929(70.01)
Rural	-	5,967(29.99)
Education		
Illiteracy	-	11,772(59.17)
Primary school or above	-	8,124(40.83)
Ethnicity		
Han	-	18,541(93.19)
Others	-	1,355(6.81)
Current smoker		
No	-	15,691(78.87)
Yes	-	4,205(21.13)
Current alcohol consumer		
No	-	15,397(77.39)
Yes	-	4,499(22.61)
Physical activity		
No	-	6,617(33.26)
Yes	-	13,279(66.74)
Self-reported health status		
Poor	-	2,112(10.62)
Fair	-	6,351(31.92)
Good	-	11,433(57.46)
History of hypertension		
No	-	8,153(40.98)
Yes	-	11,743(59.02)
History of diabetes		
No	-	19,527(98.15)
Yes	-	369(1.85)
History of cardiovascular diseases		
No	-	19,082(95.91)
Yes	-	814(4.09)
Number of natural teeth in each wave of survey		
First	9.37 ± 10.16	-
Second	8.26 ± 9.67	-
Third	8.38 ± 9.48	-
Fourth	8.68 ± 9.66	-
Fifth	4.05 ± 6.26	-
Sixth	1.92 ± 2.60	-
Seventh	1.12 ± 2.35	-
Eighth	2.20 ± 1.79	-
False teeth		
Yes	-	5,546(27.87)
No	-	14,350(72.13)
Drinking habits		
Boiled water	-	18,876(94.87)
Un-boiled water	-	1,020(5.13)
Drinking water sources at around 60 years		
Well	-	11,148(56.03)
Surface water	-	2,637(13.25)
Spring	-	749(3.76)
Tap water	-	5,362(26.95)
Staple food		
Rice	-	12,938(65.03)
Corn	-	752(3.78)
Wheat	-	3,840(19.30)
Rice and wheat		2,366(11.89)
Frequency of intake fresh fruit	-	
Almost everyday	-	2,628(13.21)
Quite often	-	3,615(18.17)
Occasionally	-	8,838(44.42)
Rarely or never	-	4,815(24.20)
Frequency of intake vegetables		
Almost everyday	-	12,836(64.52)
Quite often	-	4,357(21.90)
Occasionally	-	2,228(11.20)
Rarely or never	-	475(2.39)
Frequency of intake meat		
Almost everyday	-	9,147(45.97)
Occasionally	-	7,559(37.99)
Rarely or never	-	3,190(16.03)
Frequency of intake fish		
Almost everyday	-	5,964(29.98)
Occasionally	-	8,635(43.40)
Rarely or never	-	5,297(26.62)

Table 2 shows that differences in all baseline characteristics between included and excluded participants. Compared to the excluded participants, included participants were younger and had more natural teeth. Except history of hypertension, there were significant differences in all characteristics between included and excluded participants.

**Table 2:** The characteristics at baseline between included and excluded participants (*N* = 57,257)

Characteristics	Included participants (*n* = 19,896)	Excluded participants (*n* = 37,361)	*P*
Age (mean ± SD, years)	83.82 ± 11.13	89.19 ± 11.65	< 0.001
Weight (mean ± SD, kg)	50.54 ± 11.22	49.62 ± 12.41	< 0.001
Number of natural teeth (mean ± SD)	9.39 ± 10.17	7.81 ± 11.51	< 0.001
Sex (n, %)			< 0.001
Male	8,989(45.18)	14,788(39.58)	
Female	10,907(54.82)	22,573(60.42)	
Marital status (n, %)			< 0.001
Married	7,672(38.59)	9,984(26.85)	
Divorced	95(0.48)	152(0.41)	
Widowed	11,900(59.85)	26,672(71.72)	
Unmarried	215(1.08)	381(1.02)	
Living areas (n, %)			< 0.001
Urban	13,738(70.01)	27,270(73.64)	
Rural	5,884(29.99)	9,763(26.36)	
Education (n, %)			< 0.001
Illiteracy	11,679(58.93)	23,294(62.76)	
Primary school or above	8,141(41.07)	13,820(37.24)	
Ethnicity (n, %)			< 0.001
Han	18,292(91.94)	34,983(93.64)	
Others	1,604(8.06)	2,378(6.36)	
Current smoker (n, %)			< 0.001
No	4,200(21.12)	5,609(15.07)	
Yes	15,688(78.88)	31,594(84.91)	
Current alcohol consumer (n, %)			< 0.001
No	4,498(22.62)	6,175(16.63)	
Yes	15,389(77.38)	30,943(83.34)	
Physical activity (n, %)			< 0.001
No	6,614(33.27)	9,337(25.16)	
Yes	13,268(66.73)	27,760(74.81)	
Self-reported health status (n, %)			< 0.001
Poor	2,082(10.48)	4,745(14.78)	
Fair	6,337(31.91)	11,870(36.96)	
Good	11,442(57.61)	15,498(48.26)	
History of hypertension (n, %)			0.819
No	8,151(40.97)	15,343(41.07)	
Yes	11,745(59.03)	22,018(58.93)	
History of diabetes (n, %)			< 0.001
No	19,511(98.10)	32,053(95.07)	
Yes	377(1.90)	1,663(4.93)	
History of cardiovascular diseases (n, %)			< 0.001
No	19,063(95.86)	31,331(92.45)	
Yes	824(4.14)	2,559(7.55)	
False teeth (n, %)			< 0.001
Yes	5,630(28.31)	9,994(26.84)	
No	14,259(71.69)	27,161(72.95)	
Drinking habits (n, %)			< 0.001
Boiled water	18,881(94.96)	31,379(96.42)	
Un-boiled water	1,002(5.04)	1,145(3.52)	
Drinking water sources at around 60 years (n, %)			< 0.001
Well	10,982(56.01)	20,013(54.54)	
Surface water	2,587(13.19)	4,045(11.02)	
Spring	737(3.76)	1,181(3.22)	
Tap water	5,302(27.04)	11,190(30.50)	
Staple food (n, %)			< 0.001
Rice	12,736(64.04)	22,983(61.57)	
Corn	745(3.75)	1,415(3.79)	
Wheat	3,988(20.05)	6,995(18.74)	
Rice and wheat	2,418(12.16)	5,571(14.92)	
Frequency of intake fresh fruit (n, %)			< 0.001
Almost everyday	2,664(13.39)	6,202(16.62)	
Quite often	3,717(18.69)	7,174(19.23)	
Occasionally	8,743(43.96)	13,994(37.5)	
Rarely or never	4,765(23.96)	9,921(26.59)	
Frequency of intake vegetables (n, %)			< 0.001
Almost everyday	12,839(64.56)	22,381(59.98)	
Quite often	4,381(22.03)	8,777(23.52)	
Occasionally	2,194(11.03)	4,422(11.85)	
Rarely or never	474(2.38)	1,707(4.57)	
Frequency of intake meat (n, %)			< 0.001
Almost everyday	9,282(46.67)	22,329(59.96)	
Occasionally	7,463(37.52)	9,280(24.92)	
Rarely or never	3,144(15.81)	5,541(14.88)	
Frequency of intake fish (n, %)			< 0.001
Almost everyday	6,121(30.78)	15,910(42.73)	
Occasionally	8,540(42.94)	11,501(30.89)	
Rarely or never	5,228(26.29)	9,692(26.03)	

### Dietary habits and drinking water sources with number of natural teeth

Table 3 shows the results for the relationship between dietary habits and drinking water sources and number of natural teeth in the elderly adults. Compared with subjects drinking tap water, 1.036, 0.880 and 1.331 fewer natural teeth were reported for those drinking well (*β* = -1.036; *95 % Confidence interval (CI)*: -1.206, -0.865), surface water (*β* = -0.880; *95 % CI*: -1.122, -0.637) and spring (*β* = -1.331; *95 % CI*: -1.715, -0.947) at baseline survey. Compared with subjects with rice intake as the staple food, those with wheat intakes (*β* = -0.684; *95 % CI*: -0.865, -0.503) tended to have fewer natural teeth. Compared with participants with fresh fruit intake almost every day, those with quite often (*β* = -0.282; *95 % CI*: -0.534, -0.029), occasionally (*β* = -0.586; *95 % CI*: -0.809, -0.364), rarely or never (*β* = -1.002; *95 % CI*: -1.249, -0.755) intakes of fresh fruit tended to have fewer teeth with a significant dose-response trend (*P*_trend_ < 0.001). Similar dose-response trend was also indicated for vegetable intake (*P*_trend_ <0.001). Compared with subjects who ate meat almost every day, fewer number of teeth was found for those with less intake frequency (occasionally: *β* = -0.265; *95 % CI*: -0.425, -0.105; rarely or never: *β* = -0.236; *95 % CI*: -0.411, -0.031). Besides, fewer number of teeth was also indicated for those with less frequency of fish intake (occasionally: *β* = -0.384; *95 % CI*: -0.562, -0.207; rarely or never: *β* = -0.646; *95 % CI*: -0.840, -0.451).

**Table 3:** The associations of drinking water and dietary habits with number of natural teeth^※^

Models	*β*	*95 % CI*	*P*
**Model 1**			
Drinking habits			
Un-boiled water	*Ref*		
Boiled water	0.149	-0.158 ~ 0.457	0.342
**Model 2**			
Drinking water sources at around 60 years			
Tap water	*Ref*		
**Well**	**-1.036**	**-1.206~ -0.865**	**< 0.001**
**Surface water**	**-0.880**	**-1.122~ -0.637**	**< 0.001**
**Spring**	**-1.331**	**-1.715~ -0.947**	**< 0.001**
**Model 3**			
Staple food	*Ref*		
Rice			
Corn	-0.336	-0.689 ~ 0.018	0.063
**Wheat**	**-0.684**	**-0.865~ -0.503**	**< 0.001**
Rice and wheat	0.091	-0.127 ~ 0.309	0.413
**Model 4**			
Frequency of intake fresh fruit			
Almost everyday	*Ref*		
**Quite often**	**-0.282**	**-0.534~ -0.029**	**0.029**
**Occasionally**	**-0.586**	**-0.809~ -0.364**	**< 0.001**
**Rarely or never**	**-1.002**	**-1.249~ -0.755**	**< 0.001**
*P* for trend	-0.335	-0.411~ -0.258	< 0.001
**Model 5**			
Frequency of intake vegetables			
Almost everyday	*Ref*		
**Quite often**	**-0.595**	**-0.766~ -0.425**	**< 0.001**
**Occasionally**	**-0.505**	**-0.729~ -0.282**	**< 0.001**
**Rarely or never**	**-0.755**	**-1.224~ -0.287**	**0.002**
*P* for trend	-0.315	-0.403~ -0.226	< 0.001
**Model 6**			
Frequency of intake meat			
Almost everyday	*Ref*		
**Occasionally**	**-0.265**	**-0.425~ -0.105**	**0.001**
**Rarely or never**	**-0.236**	**-0.441~ -0.031**	**0.024**
*P* for trend	-0.143	-0.242~ -0.043	0.005
**Model 7**			
Frequency of intake fish			
**Almost everyday**	***Ref***		
**Occasionally**	**-0.384**	**-0.562~ -0.207**	**< 0.001**
**Rarely or never**	**-0.646**	**-0.840~ -0.451**	**< 0.001**
*P* for trend	-0.320	-0.417~ -0.223	< 0.001

^※^In all models, age, sex, weight, marital status, education, ethnicity, current smoker, current alcohol consumer, physical activity, living areas, false teeth, self-reported health, history of hypertension, history of diabetes, history of cardiovascular diseases, and wave were adjusted

### Subgroup analyses by sex and living areas

Tables 4 and 5 show the subgroup results of associations between dietary factors and number of natural teeth stratified by sex and living areas. Compared to subjects with rice as the staple food, significant fewer number of teeth was indicated for women with corn intake as the staple food (*β* = -0.601; *95 % CI*: -1.035, -0.167; *P* = 0.007), but not for men (*β* = -0.110; *95 % CI*: -0.693, 0.473; *P* = 0.711). Men with less frequency of meat intake was associated with fewer number of teeth (occasionally: *β* = -0.359; *95 % CI*: -0.612, -0.107; rarely or never: *β* = -0.391; *95 % CI*: -0.732, -0.050); however, such significant association was not observed in women (occasionally: *β* = -0.165; *95 % CI*: -0.368, 0.038; rarely or never: *β* = -0.146: *95 % CI*: -0.398, 0.105).

**Table 4:** The associations of drinking water and dietary habits with number of natural teeth stratified by sex^※^

Models	Males (*N* = 8,984)		Females (*N* = 10,912)	
	***P***	***β (95 % CI)***	***P***	***β (95 % CI)***
**Model 1**				
Drinking habits				
Un-boiled water	*Ref*		*Ref*	
Boiled water	0.563	0.150(-0.357 ~ 0.656)	0.522	0.124(-0.255 ~ 0.503)
**Model 2**				
Drinking water sources at around 60 years				
Tap water	*Ref*		*Ref*	
**Well**	**< 0.001**	**-1.181(-1.447~ -0.916)**	**< 0.001**	**-0.878(-1.099~ -0.658)**
**Surface water**	**< 0.001**	**-1.060(-1.456~ -0.664)**	**< 0.001**	**-0.715(-1.017~ -0.414)**
**Spring**	**< 0.001**	**-1.433(-2.038~ -0.829)**	**< 0.001**	**-1.235(-1.725~ -0.745)**
**Model 3**				
Staple food			*Ref*	
Rice	*Ref*			
Corn	0.711	-0.110(-0.693 ~ 0.473)	**0.007**	**-0.601(-1.035~ -0.167)**
**Wheat**	**< 0.001**	**-0.584(-0.871~ -0.296)**	**< 0.001**	**-0.813(-1.042~ -0.584)**
Rice and wheat	0.637	0.082 (-0.260 ~ 0.424)	0.708	0.053 (-0.226 ~ 0.332)
**Model 4**				
Frequency of intake fresh fruit				
Almost everyday	*Ref*		*Ref*	
Quite often	0.111	-0.323(-0.720 ~ 0.074)	0.175	-0.221(-0.540 ~ 0.098)
**Occasionally**	**< 0.001**	**-0.739(-1.090~ -0.388)**	**0.003**	**-0.419(-0.699~ -0.139)**
**Rarely or never**	**< 0.001**	**-1.185(-1.574~ -0.795)**	**< 0.001**	**-0.828(-1.139~ -0.518)**
*P* for trend	< 0.001	-0.402(-0.522~ -0.281)	< 0.001	-0.273(-0.369~ -0.177)
**Model 5**				
Frequency of intake vegetables				
Almost everyday	*Ref*		*Ref*	
**Quite often**	**< 0.001**	**-0.764(-1.031~ -0.496)**	**< 0.001**	**-0.482(-0.699~ -0.264)**
**Occasionally**	**0.002**	**-0.579(-0.939~ -0.220)**	**0.001**	**-0.474(-0.753~ -0.195)**
**Rarely or never**	**0.050**	**-0.807(-1.615 ~ 0.001)**	**0.012**	**-0.717(-1.275~ -0.158)**
*P* for trend	< 0.001	-0.380(-0.524~ -0.236)	< 0.001	-0.279(-0.388~ -0.169)
**Model 6**				
Frequency of intake meat				
Almost everyday	*Ref*		*Ref*	
**Occasionally**	**0.005**	**-0.359(-0.612~ -0.107)**	0.112	-0.165(-0.368 ~ 0.038)
**Rarely or never**	**0.025**	**-0.391(-0.732~ -0.050)**	0.254	-0.146(-0.398 ~ 0.105)
*P* for trend	0.005	-0.231(-0.393~ -0.068)	0.176	-0.085(-0.208 ~ 0.038)
**Model 7**				
Frequency of intake fish				
Almost everyday	*Ref*		*Ref*	
**Occasionally**	**0.029**	**-0.307(-0.584~ -0.031)**	**< 0.001**	**-0.442(-0.671~ -0.213)**
**Rarely or never**	**0.001**	**-0.531(-0.845~ -0.217)**	**< 0.001**	**-0.717(-0.961 -0.474)**
*P* for trend	0.001	-0.265(-0.423~-0.108)	< 0.001	-0.352(-0.473~ -0.231)

^※^In all models, age, weight, marital status, education, ethnicity, current smoker, current alcohol consumer, physical activity, living areas, false teeth, self-reported health, history of hypertension, history of diabetes, history of cardiovascular diseases, and wave were adjusted

**Table 5:** The associations of drinking water and dietary habits with number of natural teeth stratified by living areas^※^

Models	Urban (*N* = 13,929)		Rural (*N* = 5,967)	
	***P***	***β (95 % CI)***	***P***	***β (95 % CI)***
**Model 1**				
Drinking habits				
Un-boiled water	*Ref*		*Ref*	
Boiled water	0.085	0.323(-0.044 ~ 0.691)	0.308	-0.292(-0.852 ~ 0.269)
**Model 2**				
Drinking water sources at around 60 years				
Tap water	*Ref*		*Ref*	
**Well**	**< 0.001**	**-1.183(-1.394~ -0.971)**	**< 0.001**	**-0.606(-0.909~ -0.303)**
**Surface water**	**< 0.001**	**-1.080(-1.386~ -0.774)**	**0.042**	**-0.412(-0.811~ -0.014)**
**Spring**	**< 0.001**	**-1.267(-1.761~ -0.773)**	**< 0.001**	**-1.457(-2.065~ -0.850)**
**Model 3**				
Staple food				
Rice	*Ref*		*Ref*	
**Corn**	**0.021**	**-0.555(-1.027~ -0.082)**	0.651	-0.120(-0.638 ~ 0.399)
**Wheat**	**< 0.001**	**-0.639(-0.858~ -0.419)**	**< 0.001**	**-0.768(-1.087~ -0.449)**
Rice and wheat	0.381	-0.119(-0.385 ~ 0.147)	0.059	0.375(-0.014 ~ 0.765)
**Model 4**				
Frequency of intake fresh fruit				
Almost everyday	*Ref*		*Ref*	
Quite often	0.273	-0.175(-0.487 ~ 0.138)	0.058	-0.449(-0.914 ~ 0.016)
**Occasionally**	**0.003**	**-0.447(-0.737~ -0.158)**	**< 0.001**	**-0.735(-1.073~ -0.397)**
**Rarely or never**	**< 0.001**	**-0.975(-1.291~ -0.659)**	**< 0.001**	**-0.953(-1.342~ -0.565)**
*P* for trend	< 0.001	-0.337(-0.431~ -0.243)	< 0.001	-0.320(-0.446~ -0.193)
**Model 5**				
Frequency of intake vegetables				
Almost everyday	*Ref*		*Ref*	
**Quite often**	**< 0.001**	**-0.650(-0.843~ -0.458)**	0.115	-0.309(-0.694 ~ 0.075)
**Occasionally**	**0.004**	**-0.409(-0.690~ -0.129)**	**0.001**	**-0.623(-0.985~ -0.260)**
**Rarely or never**	**0.047**	**-0.608(-1.206~ -0.009)**	**0.014**	**-0.926(-1.661~ -0.191)**
*P* for trend	< 0.001	-0.302(-0.413~ -0.192)	< 0.001	-0.310(-0.456~ -0.164)
**Model 6**				
Frequency of intake meat				
Almost everyday	*Ref*		*Ref*	
**Occasionally**	**0.002**	**-0.307(-0.504~ -0.111)**	0.230	-0.167(-0.439 ~ 0.105)
**Rarely or never**	**0.006**	**-0.356(-0.611~ -0.102)**	0.905	0.021(-0.322 ~ 0.364)
*P* for trend	0.001	-0.202(-0.324~ -0.079)	0.876	-0.013(-0.182 ~ 0.155)
**Model 7**				
Frequency of intake fish				
Almost everyday	*Ref*		*Ref*	
**Occasionally**	**< 0.001**	**-0.569(-0.784~ -0.355)**	0.496	0.113(-0.211 ~ 0.436)
**Rarely or never**	**< 0.001**	**-0.785(-1.019~ -0.551)**	0.388	-0.159(-0.518 ~ 0.201)
*P* for trend	< 0.001	-0.388(-0.505~ -0.272)	0.266	-0.101(-0.279 ~ 0.077)

^※^In all models, age, weight, marital status, education, ethnicity, current smoker, current alcohol consumer, physical activity, living areas, false teeth, self-reported health, history of hypertension, history of diabetes, history of cardiovascular diseases, and wave were adjusted

Compared with rice as the staple food, corn intake was associated with significant fewer number of teeth for subjects living in urban area (*β* = -0.555; *95 % CI*: -1.027, -0.082), rather than those living in rural area (*β* = -0.120; *95 % CI*: -0.638, 0.399). Besides, less frequency of meat and fish intakes was associated with fewer number of teeth for subjects living in urban area (meat intake: *P*_trend_ = 0.001; fish intake: *P*_trend_ < 0.001); however, such significant association was not observed for those living in rural area (meat intake: *P*_trend_ = 0.876; fish intake: *P*_trend_ = 0.266).

### Sensitivity analysis

In the first sensitivity analysis after mutually adjusted for the drinking habits, drinking water sources at around 60 years, type of staple food, frequency of fresh fruit intake, frequency of vegetable intake, frequency of intake meat and frequency of intake fish in the multivariable models, the association disappeared for meat intake. (Table 6) In the second sensitivity analysis excluding participants with physical and cognitive impairment, the results didn’t substantially change and were consistent with the main analysis. (Table 7) In the third sensitivity analysis, the associations of drinking water and dietary habits with number of natural teeth using multiple imputation dataset were consistent with those of main analysis. (Table 8).

**Table 6:** The associations of drinking water and dietary habits with number of natural teeth in the sensitivity analysis^※^

Models	*β*	*95 % CI*	*P*
**Model 1**^**#**^			
Drinking habits			
Un-boiled water	*Ref*		
Boiled water	0.033	-0.275 ~ 0.341	0.835
**Model 2**^**#**^			
Drinking water sources at around 60 years			
Tap water	*Ref*		
**Well**	**-0.927**	**-1.099~ -0.754**	**< 0.001**
**Surface water**	**-0.896**	**-1.141~ -0.650**	**< 0.001**
**Spring**	**-1.313**	**-1.700~ -0.925**	**< 0.001**
**Model 3**^**#**^			
Staple food			
Rice	*Ref*		
Corn	-0.212	-0.568 ~ 0.144	0.243
**Wheat**	**-0.480**	**-0.669~ -0.292**	**< 0.001**
Rice and wheat	0.042	-0.178 ~ 0.263	0.708
**Model 4**^**#**^			
Frequency of intake fresh fruit			
Almost everyday	*Ref*		
Quite often	-0.075	-0.329 ~ 0.180	0.566
**Occasionally**	**-0.312**	**-0.542~ -0.083**	**0.008**
**Rarely or never**	**-0.700**	**-0.959~ -0.442**	**< 0.001**
*P* for trend	-0.245	-0.325~ -0.165	< 0.001
**Model 5**^**#**^			
Frequency of intake vegetables			
Almost everyday	*Ref*		
**Quite often**	**-0.522**	**-0.694~ -0.351**	**< 0.001**
**Occasionally**	**-0.353**	**-0.578~ -0.127**	**0.002**
**Rarely or never**	**-0.597**	**-1.068~ -0.126**	**0.013**
*P* for trend	-0.246	-0.336~ -0.156	< 0.001
**Model 6**^**#**^			
Frequency of intake meat			
Almost everyday	*Ref*		
Occasionally	-0.047	-0.215 ~ 0.122	0.586
Rarely or never	0.062	-0.165 ~ 0.288	0.593
*P* for trend	0.019	-0.092 ~ 0.129	0.740
**Model 7**^**#**^			
Frequency of intake fish			
Almost everyday	*Ref*		
**Occasionally**	**-0.284**	**-0.472~ -0.097**	**0.003**
**Rarely or never**	**-0.493**	**-0.712~ -0.274**	**< 0.001**
*P* for trend	-0.244	-0.353~ -0.135	< 0.001

^※^In all models, age, sex, weight, marital status, education, ethnicity, current smoker, current alcohol consumer, physical activity, living areas, false teeth, self-reported health, history of hypertension, history of diabetes, history of cardiovascular diseases, and wave were adjusted

^**#**^When one of drinking habits, drinking water sources at around 60 years, staple food, frequency of intake fresh fruit, frequency of intake vegetables, frequency of intake meat, frequency of intake fish, and frequency of intake egg was analyzed, other seven factor were additionally adjusted

**Table 7:** The associations of drinking water and dietary habits with number of natural teeth in the sensitivity analysis as excluding participants with physical and cognitive impairment^※^

Models	*β*	*95 % CI*	*P*
**Model 1**			
Drinking habits			
Un-boiled water	*Ref*		
Boiled water	0.186	-0.139 ~ 0.510	0.262
**Model 2**			
Drinking water sources at around 60 years			
Tap water	*Ref*		
**Well**	**-1.063**	**-1.241~ -0.885**	**< 0.001**
**Surface water**	**-0.870**	**-1.124~ -0.616**	**< 0.001**
**Spring**	**-1.391**	**-1.793~ -0.989**	**< 0.001**
**Model 3**			
Staple food			
Rice	*Ref*		
**Corn**	**-0.434**	**-0.795~ -0.074**	**0.018**
**Wheat**	**-0.731**	**-0.922~ -0.541**	**< 0.001**
Rice and wheat	0.101	-0.127 ~ 0.330	0.385
**Model 4**			
Frequency of intake fresh fruit			
Almost everyday	*Ref*		
**Quite often**	**-0.286**	**-0.548~ -0.024**	**0.032**
**Occasionally**	**-0.624**	**-0.852~ -0.397**	**< 0.001**
**Rarely or never**	**-0.972**	**-1.227~ -0.718**	**< 0.001**
*P* for trend	-0.328	-0.407~ -0.249	< 0.001
**Model 5**			
Frequency of intake vegetables			
Almost everyday	*Ref*		
**Quite often**	**-0.613**	**-0.793~ -0.432**	**< 0.001**
**Occasionally**	**-0.501**	**-0.734~ -0.267**	**< 0.001**
**Rarely or never**	**-0.864**	**-1.358~ -0.369**	**0.001**
*P* for trend	-0.326	-0.419~ -0.234	< 0.001
**Model 6**			
Frequency of intake meat			
Almost everyday	*Ref*		
**Occasionally**	**-0.284**	**-0.449~ -0.118**	**0.001**
**Rarely or never**	**-0.272**	**-0.485~ -0.058**	**0.013**
*P* for trend	-0.161	-0.264~ -0.057	0.002
**Model 7**			
Frequency of intake fish			
Almost everyday	*Ref*		
**Occasionally**	**-0.346**	**-0.531~ -0.161**	**< 0.001**
**Rarely or never**	**-0.598**	**-0.803~ -0.394**	**< 0.001**
*P* for trend	-0.297	-0.399~ -0.195	< 0.001

^※^In all models, age, sex, weight, marital status, education, ethnicity, current smoker, current alcohol consumer, physical activity, living areas, false teeth, self-reported health, history of hypertension, history of diabetes, history of cardiovascular diseases, and wave were adjusted

**Table 8:** The associations of drinking water and dietary habits with number of natural teeth using multiple imputation dataset^※^

Models	*β*	*95 % CI*	*P*
**Model 1**			
Drinking habits			
Un-boiled water	*Ref*		
Boiled water	0.110	-0.056 ~ 0.275	0.195
**Model 2**			
Drinking water sources at around 60 years			
Tap water	*Ref*		
**Well**	**0.192**	**0.066 ~ 0.318**	**0.004**
Surface water	0.074	-0.175 ~ 0.323	0.565
**Spring**	**0.408**	**0.272 ~ 0.545**	**< 0.001**
**Model 3**			
Staple food			
Rice	*Ref*		
Corn	-0.203	-0.466 ~ 0.060	0.150
**Wheat**	**-0.490**	**-0.624~ -0.357**	**< 0.001**
**Rice and wheat**	**-0.196**	**-0.340~ -0.053**	**0.012**
**Model 4**			
Frequency of intake fresh fruit			
Almost everyday	*Ref*		
Quite often	-0.085	-0.242 ~ 0.072	0.292
**Occasionally**	**-0.223**	**-0.350~ -0.097**	**0.001**
**Rarely or never**	**-0.449**	**-0.613~ -0.286**	**< 0.001**
**Model 5**			
Frequency of intake vegetables			
Almost everyday	*Ref*		
**Quite often**	**-0.327**	**-0.426~ -0.229**	**< 0.001**
**Occasionally**	**-0.175**	**-0.307~ -0.043**	**0.013**
**Rarely or never**	**-0.293**	**-0.497~ -0.089**	**0.005**
**Model 6**			
Frequency of intake meat			
Almost everyday	*Ref*		
**Occasionally**	**-0.153**	**-0.264~ -0.043**	**0.012**
**Rarely or never**	**-0.241**	**-0.371~ -0.111**	**0.001**
**Model 7**			
Frequency of intake fish			
**Almost everyday**	***Ref***		
**Occasionally**	**-0.283**	**-0.388~ -0.177**	**< 0.001**
**Rarely or never**	**-0.408**	**-0.515~ -0.302**	**< 0.001**

^※^In all models, age, sex, weight, marital status, education, ethnicity, current smoker, current alcohol consumer, physical activity, living areas, false teeth, self-reported health, history of hypertension, history of diabetes, history of cardiovascular diseases, and wave were adjusted

## Discussion

In this longitudinal study, we explored the effect of dietary intake/water sources on tooth loss. The exposure were dietary intake or water sources, and the outcome of interest was tooth loss. We found significantly fewer number of teeth associated with drinking well, surface water, and spring, intakes of wheat as staple food, as well as less frequency of fresh fruit, vegetable, meat and fish intakes. Furthermore, corn intake as the staple food, and less frequency of meat and fish intakes was associated with fewer number of teeth for subjects living in urban area. Besides, fewer number of teeth was observed in men who had less frequency of meat intake, and in women who ate corn as the stale food. This longitudinal study might help illustrate the temporality and potential causality regarding the effect of dietary intake and drinking water on the number of natural teeth.

Several cross-sectional studies have explored the relation of impaired dentition status with poor nutritional intake. In the early 2000 s, large cross-sectional studies on the relationship between nutritional and oral status were conducted in Britain and the United States and Japan, and found possible relationship of tooth loss with fruit, vegetable, and vitamin intake. [18] In another large cross-sectional study, the number of teeth present had a significant relationship with the intake of several nutrients. In particular, total protein, animal protein, sodium, vitamin D, vitamin B1, vitamin B6, niacin, and pantothenic acid were significantly associated with the number of teeth present. [7] The intake of vegetables, fish, and shellfish was significantly lower among subjects with fewer teeth. [7] These studies suggested a possible link between tooth loss and dietary intake, which was in line with the present study. However, since the nature of the cross-sectional design, they were unable to clarify the temporal sequence and failed to imply a causal association.

Of note, previous longitudinal studies mainly focused on the effects of tooth loss on dietary and nutritional intake. [19] In 2017, a systematic review of eight longitudinal studies was conducted to explore whether tooth loss affects dietary intake and nutritional status among adults. [19] Most studies investigating the association between tooth loss and dietary intake and nutritional status showed significant results. A consistent association was observed between greater tooth loss and smaller reductions in dietary cholesterol. This review suggested a potential effect of tooth loss on diet and nutrition. However, few prospective cohort studies have evaluated the opposite direction of the association, i.e., the effect of dietary intakes on teeth loss. A small longitudinal study of 57 elderly Japanese subjects suggested that there was a significant relationship between nutrient intake, such as minerals and vitamins from food, and tooth loss. [7] The lower intake of vegetables and fish, shellfish, and their products was significantly associated with fewer teeth. [7] This study further supported our current findings that fewer number of teeth was associated with less frequency of vegetable and fish intakes in Chinese elderly subjects.

Dental disease has been widely recognized as one of the main causes of tooth loss. Caries is the most common dental disease, and sugar plays a decisive role in the occurrence of caries. [20] Sucrose can be used by cariogenic bacteria to accelerate its growth. [21] The bacteria on the teeth, such as streptococcus mutans, uses sugars in our diet as a source of energy, which may product acid and result in tooth decay. [22, 23] Besides, the texture and physical properties of food will affect the residence of food on the tooth surface. Of note, periodontal diseases such as periodontitis is another main cause of tooth loss. [24, 25] Related studies have found that chewing food can wash and massage gingiva, which is beneficial to the health of periodontal tissue and reduce tooth loss. Therefore, different eating frequency may affect number of teeth through periodontal disease. Regarding the source of drinking water, it is interesting that different water types may affect dental health. Compared with tap water, well, surface water or spring were associated with fewer teeth in the elderly. It is generally hypothesized that the additions of fluoride to tap water may provide strong benefits on a person’s oral health. [10, 26, 27] However, the situation in China is unique, and there are wide variations of fluoride levels in drinking water in China. Guangdong Province is the only province that has experienced artificial water fluoridation in China during 1965 to 1983. [10] Besides, there is no clear evidence that the tap water in China contains higher level of fluoride than other water sources in other areas of China. Thus, further cohort studies are still warranted to explore the relationship between different water sources and tooth loss.

The major strength of this study is prospective cohort design, which may enable us to explore the temporal relationship between dietary factors and number of natural teeth. However, there are also some limitations. First, the recall of the dietary intake in the elderly may be not accurate. Thus, we may fail to capture the accumulated intakes during a specific time period. This may also lead to recall bias and misclassification bias. Second, the number of natural teeth was self-reported by older adults, but not be checked by research staff. Due to the self-reported nature, the number of teeth may be not accurate. However, previous validity studies indicated that self-reports were considered valid alternatives to clinical measures to estimate tooth counts in adult population. [28, 29] Third, although we have adjusted multiple important risk factors in the models, we still cannot rule out the possibility of residual or unmeasured confounding. For example, the total energy intake could not be adjusted in the model because this variable cannot be derived from the current dietary questionnaires. Moreover, we did not consider other beverages intakes, especially sugar-sweetened beverages in the analysis, which may also lead to residual confounding to current results. Fourth, this study was conducted in the Chinese elderly subjects, thus, the generalization of the results to younger population or other study population should be cautious. Lastly, the number of teeth affects soft foods and hard foods consumed. However, food texture was not mentioned in the CLHLS. Therefore, the influence of food texture can’t be corrected.

## Conclusions

There are significantly fewer number of teeth associated with drinking well, surface water, and spring compared with tap water in Chinese elderly population. Moreover, intakes of wheat as staple food, as well as less frequency of fresh fruit, vegetables, meat and fish intakes were also associated with fewer number of teeth in the elderly adults. Further large-scale, well-designed cohort studies are still warranted to conform the findings. If these associations were proved to be causal, future dietary recommendations could consider the impact of diet on both the general health and the oral health.

## Supplementary information

Additional file 1 Supplementary document Questionnaire for the Chinese Longitudinal Healthy Longevity Survey.

## Data Availability

All the datasets analyzed in this study are available in the website of the CLHLS (https://opendata.pku.edu.cn/dataverse/CHADS).

## Abbreviations

CLHLS: Chinese Longitudinal Healthy Longevity Survey
CI: Confidence interval

## References

[1] Watanabe Y, Okada K, Kondo M, Matsushita T, Nakazawa S, Yamazaki Y (2020). Oral health for achieving longevity. Geriatr Gerontol Int.

[2] Dye B, Thornton-Evans G, Li X, Iafolla T: **Dental caries and tooth loss in adults in the United States, 2011–2012**. *NCHS Data Brief* 2015(197):197. 25973996

[3] Vedin O, Hagstrom E, Ostlund O, Avezum A, Budaj A, Flather MD, Harrington RA, Koenig W, Soffer J, Siegbahn A (2017). Associations between tooth loss and prognostic biomarkers and the risk for cardiovascular events in patients with stable coronary heart disease. Int J Cardiol.

[4] Shakeri R, Malekzadeh R, Etemadi A, Nasrollahzadeh D, Abedi-Ardekani B, Khoshnia M, Islami F, Pourshams A, Pawlita M, Boffetta P (2013). Association of tooth loss and oral hygiene with risk of gastric adenocarcinoma. Cancer Prev Res (Phila).

[5] Komagamine Y, Kanazawa M, Iwaki M, Jo A, Suzuki H, Amagai N, Minakuchi S (2016). Combined effect of new complete dentures and simple dietary advice on nutritional status in edentulous patients: study protocol for a randomized controlled trial. Trials.

[6] Hung HC, Willett W, Ascherio A, Rosner BA, Rimm E, Joshipura KJ (2003). Tooth loss and dietary intake. J Am Dent Assoc.

[7] Yoshihara A, Watanabe R, Nishimuta M, Hanada N, Miyazaki H (2005). The relationship between dietary intake and the number of teeth in elderly Japanese subjects. Gerodontology.

[8] **From the Centers for Disease Control and Prevention. Achievements in public health, 1900–1999: fluoridation of drinking water to prevent dental caries**. *JAMA* 2000, **283**(10):1283–1286. 10.1001/jama.283.10.1283 10714718

[9] O’Mullane DM, Baez RJ, Jones S, Lennon MA, Petersen PE, Rugg-Gunn AJ, Whelton H, Whitford GM (2016). Fluoride and Oral Health. Community Dent Health.

[10] Petersen PE, Kwan S, Zhu L, Zhang BX, Bian JY (2008). Effective use of fluorides in the People’s Republic of China–a model for WHO Mega Country initiatives. Community Dent Health.

[11] Tungare S, Paranjpe AG: **Diet and Nutrition To Prevent Dental Problems**. In: *StatPearls.* Treasure Island (FL); 2021. 30480981

[12] Christensen K, Thinggaard M, Oksuzyan A, Steenstrup T, Andersen-Ranberg K, Jeune B, McGue M, Vaupel JW (2013). Physical and cognitive functioning of people older than 90 years: a comparison of two Danish cohorts born 10 years apart. Lancet.

[13] Shi Z, Zhang T, Byles J, Martin S, Avery JC, Taylor AW (2015). Food Habits, Lifestyle Factors and Mortality among Oldest Old Chinese: The Chinese Longitudinal Healthy Longevity Survey (CLHLS). Nutrients.

[14] Zeng Y, Feng Q, Hesketh T, Christensen K, Vaupel JW (2017). Survival, disabilities in activities of daily living, and physical and cognitive functioning among the oldest-old in China: a cohort study. Lancet.

[15] Feng T, Feng Z, Liu Q, Jiang L, Yu Q, Liu K (2021). Drinking habits and water sources with the incidence of cognitive impairment in Chinese elderly population: The Chinese Longitudinal Healthy Longevity Survey. J Affect Disord.

[16] Yang HL, Li FR, Chen PL, Cheng X, Chen M, Wu XB: **Tooth Loss, Denture Use and Cognitive Impairment in Chinese Older Adults: A Community Cohort Study**. *J Gerontol A Biol Sci Med Sci* 2021. 10.1155/2021/6155304 PMC8497166 33674815

[17] Li Q, Ge YL, Li M, Fang XZ, Yuan YP, Liang L, Huang SQ (2017). miR-127 contributes to ventilator-induced lung injury. Molecular medicine reports.

[18] Yoshida M, Suzuki R, Kikutani T (2014). Nutrition and oral status in elderly people. Japanese Dental Science Review.

[19] Gaewkhiew P, Sabbah W, Bernabe E (2017). Does tooth loss affect dietary intake and nutritional status? A systematic review of longitudinal studies. J Dent.

[20] Rathee M, Sapra A: **Dental Caries**. In: *StatPearls.* Treasure Island (FL); 2021.

[21] Rezende G, Arthur RA, Grando D, Hashizume LN (2017). Cariogenic Potential of Sucrose Associated with Maltodextrin on Dental Enamel. Caries Res.

[22] Costalonga M, Herzberg MC (2014). The oral microbiome and the immunobiology of periodontal disease and caries. Immunol Lett.

[23] Lemos JA, Palmer SR, Zeng L, Wen ZT, Kajfasz JK, Freires IA, Abranches J, Brady LJ: **The Biology of Streptococcus mutans**. *Microbiol Spectr* 2019, **7**(1). 10.1128/microbiolspec.gpp3-0051-2018 PMC6615571 30657107

[24] Mehrotra N, Singh S: **Periodontitis**. In: *StatPearls.* Treasure Island (FL); 2021.

[25] Gasner NS, Schure RS: **Periodontal Disease**. In: *StatPearls.* Treasure Island (FL); 2021.

[26] Neidell M, Herzog K, Glied S (2010). The association between community water fluoridation and adult tooth loss. Am J Public Health.

[27] Horowitz HS (1996). The effectiveness of community water fluoridation in the United States. J Public Health Dent.

[28] Ueno M, Shimazu T, Sawada N, Tsugane S, Kawaguchi Y (2018). Validity of self-reported tooth counts and masticatory status study of a Japanese adult population. J Oral Rehabil.

[29] Ramos RQ, Bastos JL, Peres MA (2013). Diagnostic validity of self-reported oral health outcomes in population surveys: literature review. Rev Bras Epidemiol.

