# Correction to: Associations of dietary and drinking water habits with number of natural teeth: a longitudinal study in the Chinese elderly population

**DOI:** 10.1186/s12877-021-02562-7

**Published:** 2022-03-18

**Authors:** Dan Zhao, Jia Ning, Yifei Zhao, Eryi Lu

**Affiliations:** 1 Department of Stomatology, Renji Hospital, School of Medicine, Shanghai Jiao Tong University, Shanghai, 200127 China; 2 School of Stomatology, Tianjin Medical University, No 22. Qixiangtai Road, Heping District, Tianjin, 300070 China

**Correction to: BMC Geriatr 21, 525 (2021)**

**https://doi.org/10.1186/s12877-021-02473-7**

During the publication process of the original article [1] the following contribution statement was accidentally omitted:

- Dan Zhao & Jia Ning contributed equally to this work

The publisher apologizes for the inconvenience caused.
