# Supplementary material for: Associations of dietary and drinking water habits with number of natural teeth: a longitudinal study in the Chinese elderly population
Source: BMC Geriatr. 2021 Oct 2;21:525. doi: 10.1186/s12877-021-02473-7 (PMC8487487; doi:10.1186/s12877-021-02473-7)
Supplement: Supplementary file 1 — Supplementary document Questionnaire for the Chinese Longitudinal Healthy Longevity Survey. [file 12877_2021_2473_MOESM1_ESM.pdf]

Interviews are based on voluntary participation

Questionnaire for Interviews to the Surviving Participants in Chinese Longitudinal Healthy Longevity Survey

(Approved by National Statistics Bureau, China, P. R. NSB Doc. No.: 2014(0004)

Notes:
(1) The shaded questions about characteristics that do not change such as education, occupation before retirement, childhood conditions and information about the history of the life course will not be asked again in the follow-up interviews for those previously recruited participants.
(2) As compared with CLHLS previous waves' questionnaires, several questions were newly added PhenX measures closely related to healthy aging. They will be asked for all interviewees including previously and newly recruited participants.

Guarantee for interviewee

All individual information collected in this survey will be treated as strictly confidential. The record of your name and address will be used only in future follow-up surveys to enable us to contact with you. The computerized data resulting from this survey will not include your name and address. So, nobody will be able to identify any interviewee from the computerized data files. All of the questionnaires will be stored in the locked files containers.

Questionnaire NO. □□□□
Province □□
County or city □□□□
Category of sampled elder: □
(A) centenarians (B) nonagenarians (C) octogenarians (D) age 65-79

Interviewee's name:___________________
Current Address: ____
detailed village or street address (including street, apartment #, etc.)
_______________ ____________ _________
district or township county or city province

Post Code: □ □ □ □ □ □ Tel No:________ ___________________
(area code)

Contact person: __________________________

Interview Record

Date and time of interview				Reasons for not finishing questionnaire			
month	day	start time	end time	1 the interviewee refused to be interviewed	2 the interviewee has died	3 the interviewee has emigrated	4 other
□□	□□						□

Signature

Interviewer: ____________________________; 1st check at provincial level__________________ ;

2nd check at provincial level _______________; Final check at provincial level________________;

1st check in Beijing _______________________; 2nd check in Beijing _________________________ ;

Category of interviewee

I. Category of the interviewee: ☐

1. Elder who was interviewed in all previous waves (1998, 2000, 2002, 2005, 2008/2009, and 2011/2012);
2. Elder who was interviewed in last five waves (2000, 2002, 2005, 2008/2009, and 2011/2012);
3. Elder who was interviewed in last four waves (2002, 2005, 2008/2009, and 2011/2012);
4. Elder who was interviewed in last three waves (2005, 2008/2009, and 2011/2012)
5. Elder who was interviewed in last two waves (2008/2009 and 2011/2012)
6. Elder who was interviewed in 2011/2012 only
7. Newly added elder to replace a deceased, migrated, refusal, or lost to follow-up interviewee;

   Identification code of the previously interviewed elder: ☐☐☐☐☐☐
8. Sibling aged 80+ of another interviewee aged 80+

If answer is not 6, please skip to III

II. If (s)he is a sibling of an interviewed elder (name______), what is the identification code of that interviewed elder?

☐☐☐☐☐☐

Please specify what kind of sibling ☐

1 sibling 2 half sibling 3 adopted sibling 4 others

III. current residence area of interviewee ☐

1 city 2 town 3 rural

IV. validated age (See H5) ☐☐☐

General Instructions to interviewer

1. *All questions marked with a '*' must be answered, if possible. The answers must come from the interviewees themselves, i.e., these questions may not be answered by other people. If the interviewee is not able to answer a question, please circle 'not able to answer'. In addition, interviewers should encourage the elders to attempt to answer as many of the other questions (those without a '*') as possible.*
2. *If some of the questions without a '*' cannot be answered by the elders themselves, interviewers should ask their closest relative(s) or caregiver(s) to answer them. (The box ○ in the third column is to be checked whenever the question is not answered by the interviewee.) If no one can answer the question, please explain why in the margin.*
3. *Detailed instructions (including illustrations of definitions, how to measure, examples, etc.) are presented in the interviewers instruction booklet (in easily understandable Chinese language).*
4. *Interviewees who newly join the survey should attempt to answer all questions, including those appearing on a shaded background. Those who were interviewed in the previous*

survey should attempt to answer only the questions that do not appear on a shaded background.

**A. BASIC INFORMATION**			Code
A1 Sex	1 male 2 female	○	□
A2 Ethnic group	1 Han 2 Hui 3 Zhuang 4 Yao<br>5 Korean 6 Manchu 7 Mongolia<br>8 Other	○	□
A3 Current Age	_______		□□□
A3.1 Animal year of interviewee's birth	1 rat 2 ox 3 tiger 4 rabbit<br>5 dragon 6 snake 7 horse<br>8 sheep 9 monkey 10 rooster<br>11 dog 12 boar	○	□□
A3.2 Date of birth:<br>(a) Western calendar<br>(b) Chinese calendar	 year________ month____<br>year________ month____	○<br>○	□□□□ □□<br>□□□□ □□
A4.1 In which province were you born?	province __________	○	□□
A4.2 In which county (city) were you born?	1 same as current address<br>2 other city or county	○	□
A4.3 Was the place of birth an urban area or a rural area (at time of birth)?	1 urban 2 rural	○	□
A5.1 Co-residence	1 with household member(s)<br>2 alone---*skip to A5.3.3*<br>3 in a nursing home---*skip to A5.4*	○	□
A5.2 How many people are living with you? (excluding yourself)	_______ person(s)	○	□□

Current Environmental Tobacco Smoke Exposure (Phenx Code: 060700)

A5.3 Other members of your household	Relationship with interviewee	name	sex	age	educa-tion	Cigarettes smoked per day inside home		relatio-nship	age	Sex	educat-ion
(Use back of paper for additional people, if necessary.)<br>*If age is unknown, please fill in '888'.*<br>Relationship with interviewee:<br>0 spouse<br>1 child<br>2 spouse of child<br>3 grandchild<br>4 spouse of grandchild<br>5 great grandchild or spouse of great grandchild<br>6 sibling<br>7 parent or parent-in-law<br>8 other ____________<br>Sex: 1 male 2 female<br>Education level:<br>1 Never attended school<br>2 Primary school<br>3 Middle school<br>4 High school<br>5 Associate college<br>Number of cigarettes he/she usually smoke per day inside home:<br>If unknown, please fill in "88".							○	□	□□□	□	□
							○	□	□□□	□	□
							○	□	□□□	□	□
							○	□	□□□	□	□
							○	□	□□□	□	□
							○	□	□□□	□	□
							○	□	□□□	□	□
							○	□	□□□	□	□
							○	□	□□□	□	□
							○	□	□□□	□	□
							○	□	□□□	□	□
							○	□	□□□	□	□

A5.3.0 Is your house/apartment purchased/self-built/inherited/ rented?	1 purchased 2 self-built<br>3 inherited<br>4 welfare-oriented public housing<br>5 rented or subleased<br>6 others_________	○	□
A5.3.1 Under whose name was your current house/apartment purchased/self-built/inherited?	1 self or spouse 2 child(ren)<br>3 grandchild(ren)<br>4 relative(s) 5 other	○	□
A5.3.2 Do you (and your spouse) have your own bedroom?	1 yes 2 no	○	□
**Characteristics of Current Residence** (PhenX code: 060100)			
A5.3.3 What type of dwelling is your home?	1 Detached house<br>2 Duplex/Triplex<br>3 Row house<br>4 Low rise apartment (1-3 floors)<br>5 High rise apartment (>3 floors)<br>6 Mobile home / Trailer<br>7 other	○	□
A5.3.4 What is the approximate year your home was built?	Year built ___________	○	□□□□
A5.3.5 During the past 12 months, has there been water or dampness in your home from broken pipes, leaks, heavy rain, or floods?	1 yes 2 no<br>8 don't know	○	□

A5.3.6 Does your home frequently have a mildew odor or musty smell?	1 yes 2 no<br>8 don't know	○	□
A5.3.7 Which fuels are normally used for cooking in your home?	0 Never cooking<br>1 Gas: from underground pipes serving the neighborhood<br>2 Gas: bottled, tank, or LP<br>3 Electricity<br>4 Fuel oil, kerosene, etc<br>5 Coal or coke<br>6 Charcoal<br>7 Solar energy<br>8 Firewood or straw<br>9 other<br>99 Don't know	○	□
If living with family, skip to Part B;<br>*If living alone, skip to A5-4*			
A5.4.0 What is the primary reason that you live in an institution (Elderly center, elderly home, welfare center, etc.)	1 no child or child is unavailable for caregiving<br>2 don't want to bother children<br>3 no own house/apartment but want to separate with children<br>4 able to interact with other old persons<br>5 others_____	○	□
A5.4.1 What is the average monthly cost for you living in an institution?	____________ (Yuan) (if more than 10,000, please code 9998)	○	□□□□
A5.4.2 Who mainly pays the cost?	1 self 2 spouse<br>3 children & their spouses<br>4 grandchildren & their spouses<br>5 state/collectives 6 others	○	□
A5.4 If living alone or in nursing home, since when?	Year________ month _______	○	□□□□ □□

**B.** **LIFE EVALUATION AND PERSONALITY** **(**to be answered by interviewee **ONLY)**			Code
**B1. Life Satisfaction and Self-Rated Health**			
* B1.1 How do you rate your life at present?	1 very good 2 good<br>3 so so 4 bad<br>5 very bad<br>8 not able to answer		□
* B1.2 How do you rate your health at present?	1 very good 2 good<br>3 so so 4 bad<br>5 very bad<br>8 not able to answer		□

* B1.2.1 How do you rate your health at present compared with one year ago?	1 much better 2 slightly better<br>3 almost the same 4 slightly worse<br>5 much worse<br>8 not able to answer		☐
**B2. Personality**			
* B2.1 Do you always look on the bright side of things?	1 always 2 often 3 sometimes<br>4 seldom 5 never<br>8 not able to answer		☐
* B2.2 Do you like to keep your belongings neat and clean?	1 always 2 often 3 sometimes<br>4 seldom 5 never<br>8 not able to answer		☐
* B2.3 Do you often feel fearful or anxious?	1 always 2 often 3 sometimes<br>4 seldom 5 never<br>8 not able to answer		☐
* B2.4 Do you often feel lonely and isolated?	1 always 2 often 3 sometimes<br>4 seldom 5 never<br>8 not able to answer		☐
* B2.5 Can you make your own decisions concerning your personal affairs?	1 always 2 often 3 sometimes<br>4 seldom 5 never<br>8 not able to answer		☐
* B2.6 Do you feel the older you get, the more useless you are?	1 always 2 often 3 sometimes<br>4 seldom 5 never<br>8 not able to answer		☐
* B2.7 Are you as happy as when you were younger?	1 same 2 often 3 sometimes<br>4 seldom 5 never<br>8 not able to answer<br>*(If you feel happier than when you were young, please fill in '1')*		☐
**Depression** (PhenX code: 120500)			
* B2.8 Have you had a time in last 12 months when you felt sad, blue, or depressed for two weeks or more?	1 Yes 2 No<br>8 not able to answer		☐
* B2.9 Have you had a time in last 12 months lasting two weeks or more when you lost interest in most things like hobbies, work, or activities that usually give you pleasure?	1 Yes 2 No<br>8 not able to answer		☐
If answer "No" to B2.8 and B2.9, skip to Part C<br>*If answer "YES" to either of the B2.8 or B2.9, continue to B2.10*			
* B2.10 How much of the day did these feelings usually last?	1 All day long<br>2 Most of the day<br>3 About half of the day<br>4 Less than half of the day<br>8 not able to answer		☐

C. MINI MENTAL STATE EXAMINATION (MMSE)			Code
C1 ORIENTATION (to be answered by interviewee **ONLY**)			
* C1.1 What time of day is it right now (morning, afternoon, evening)?	1 correct 0 wrong<br>8 not able to answer		☐
* C1.2 What is the month (Western or Chinese calendar) right now?	1 correct 0 wrong<br>8 not able to answer		☐
* C1.3 What is the date (Chinese calendar day and month) of the mid-autumn festival?	1 correct 0 wrong<br>8 not able to answer		☐
* C1.4 What is the season right now, spring, summer, fall, winter?	1 correct 0 wrong<br>8 not able to answer		☐
* C1.5 What is the name of this county or district?	1 correct 0 wrong<br>8 not able to answer		☐
* C1.6 Please name as many kinds of food as possible in 1 minute.	_______ (kinds of food)		☐☐
C2 REGISTRATION (to be answered by interviewee **ONLY**)			
* C2.1 I am now going to test your memory. I will mention three objects.<br>*(Mention the following three objects without pausing:)*<br>table, apple, clothes<br>Please repeat these three objects.<br>*(Evaluation based on first attempt only.)*<br>*---- If all three questions are answered correct, please skip to C3.1*<br>table<br>apple<br>clothes	1 correct 0 wrong<br>8 not able to answer<br><br>_______<br>_______<br>_______		☐<br>☐<br>☐
*C2.2 *If answers are insufficient or incorrect on the first attempt, repeat the names of all objects until the interviewee is able to name all three of them (6 attempts at maximum). Write the number of attempts (e.g., '1', if all three objects are repeated correctly on first attempt). Write '7' if interviewee cannot repeat the names even after 6 attempts.*	________attempts		☐
C3 ATTENTION AND CALCULATION (to be answered by interviewee **ONLY**)			

* C3.1 I will ask you to spend 3 dollars from 20 dollars, then you must spend 3 dollars from the number you arrived at and continue to spend 3 dollars until you are asked to stop.			
(Circle '1' each time the difference is 3 – even if a former answer was wrong. If the interviewee forgets the last number, the interviewer should repeat it, but then circle '0' even if the answer was correct. Maximum score = 5 correct answers.)	1st 1 correct 0 wrong<br>8 not able to answer<br>2nd 1 correct 0 wrong<br>8 not able to answer<br>3rd 1 correct 0 wrong<br>8 not able to answer<br>4th 1 correct 0 wrong<br>8 not able to answer<br>5th 1 correct 0 wrong<br>8 not able to answer		☐<br>☐<br>☐<br>☐<br>☐
* C3.2 Ask the interviewee to draw the figure on B Card.<br>*(Circle '1' if all the sides and angles are correct and if the figure in the middle is a quadrangle. Otherwise, circle '0'.)*	1 correct 0 wrong<br>8 can't use pen to draw the figure<br>9 not able to do this (disabled)		☐
C4 RECALL (to be answered by interviewee **ONLY**)			
* C4.1 Please repeat the three words (in any order) that I asked you to repeat a little while ago.<br>(Note the correct or the wrong answers as the scores.)<br>table<br>apple<br>clothes	1 correct 0 wrong<br>8 not able to do this<br>______<br>______<br>______		☐<br>☐<br>☐
C5 LANGUAGE (to be answered by interviewee **ONLY**)			
* C5.1 *Give the interviewee a pen and then a watch and ask what these objects are called (1 point for each correct answer).*<br>pen<br>watch	1 correct 0 wrong<br>8 not able to answer<br>_______<br>_______		☐<br>☐

* C5.2 I will now ask you to repeat the following sentence:<br>'What you plant, what you will get.'<br>*(Circle '1' only if repeated correctly on the first attempt.)*	1 correct 0 wrong<br>8 not able to answer		☐
* C5.3 I will give you a piece of paper. You must take the paper using your right hand, fold it in the middle using both hands, and place the paper on the floor.<br>*(Read the text aloud and hand a piece of paper to the interviewee. Give the paper directly to the interviewee. Do not repeat the instructions and do not offer any help. Note every movement as correct if it is made in the correct order.)*	1 correct 0 wrong<br>8 not able to do		
right hand	_______		☐
folding	_______		☐
on the floor	_______		☐
(Questions C5.4 and C5.5 are for Interviewer ONLY)			
C5.4 Was the interviewee able to answer the questions in sections B and C?	1 yes --- Skip *to session D*<br>2 no 3 partly		☐
C5.5 If 'no' or 'partly', what is the main reason?	1 visually impaired, but can hear<br>2 hearing impaired, but can see<br>3 visually and hearing impaired<br>4 paralyzed<br>5 did not wish to participate<br>6 could not understand because of cognitive impairment<br>7 not able to participate at the moment because of some temporary illness such as a cold<br>8 could not understand question<br>9 other (please explain):<br>________________________		☐

**D. LIFE STYLE**	Code

D1 Please tell us the staple food you eat.		1 rice<br>2 corn (maize)<br>3 wheat (noodles, bread, etc.)<br>4 half rice and half wheat<br>5 other:_______________		○		☐	
D2 How much of the above food do you normally eat per day?		________ liang		○		☐☐	
D3.1 Do you eat fresh fruit?		1 everyday or almost everyday<br>2 quite often<br>3 occasionally<br>4 rarely or never		○		☐	
D3.2 Do you eat fresh vegetables?		1 everyday or almost everyday<br>2 quite often<br>3 occasionally<br>4 rarely or never		○		☐	
D3.3 What kind of grease do you mainly use for cooking?		1 vegetable grease<br>2 gingili grease<br>3 lard<br>4 other animal's fat				☐	
D3.4 what kind of flavor do you mainly have?		1 Insipidity 2 Salty 3 Sweet<br>4 Hot 5 Crude<br>6 Do not have all the above tastes				☐	
D4 Please tell me what other kinds of food you normally eat and how often.		around age 60	at present		around age 60	at present	
1 almost every day<br>2 occasionally<br>3 rarely or never	meat			○	☐	☐	
	fish			○	☐	☐	
	eggs			○	☐	☐	
	food made from beans (tofu, etc.)			○	☐	☐	
	salt-preserved vegetables			○	☐	☐	
	sugar			○	☐	☐	
	tea			○	☐	☐	
	garlic			○	☐	☐	
	Milk products			○	☐	☐	
	Nut (peanut, walnut, etc)			○	☐	☐	
	Mushroom or algae			○	☐	☐	

Vitamins (A/C/E,) products			○	□	□
Medicinal plant			○	□	□
	around age 60	at present		around age 60	at present
D4.a What type of tea did you mainly drink at present?<br>D4.b What type of tea did you mainly drink around age 60?<br>1. Green tea; 2. Red tea (black tea);<br>3. Oolong tea; 4. White tea;<br>5. Yellow tea; 6. Dark tea (Pu-erh tea);<br>7. Compressed tea; 8. Scented tea;<br>9. Others, please specify:					
D5 What kind of water do you usually drink?	1 boiled water 2 un-boiled water		○	□	

D6 Such water is (was):	childhood	around age 60	at present	○	childhood	age 60	present
1 water from a well<br>2 water from a river or lake<br>3 water from a spring<br>4 water from a pond or pool<br>5 tap water				○	□	□	□

D7.1 Do you smoke at the present time?	1 yes 2 no. *If "No", skip to D7.8*	○	□
D7.2 Did you smoke in the past?	1 yes 2 no	○	□
If the answers of D71 is 'No', please skip to D7.8			
D7.3 How old were you when you began to smoke?	age _______	○	□□□
D7.4 How old were you when you stopped smoking if you don't smoke at present?	age _______<br>*If still smoking now, please code 999;If don't remember when stopped, please code 888.*	○	□□□
D7.5 If you smoke at the present, how many times per day on average do you smoke?	times _______	○	□□
**Tobacco - Nicotine Dependence** (PhenX code: 031001)			
D7.6 How soon after you wake up do you smoke your first cigarette?	1 Within 5 minutes<br>2 6-30 minutes<br>3 31-60 minutes<br>4 After 60 minutes	○	□
**Personal Perception and Knowledge of Smoking-related Cancer Risk** (PhenX code: 070700)			

D7.7 Compared to others your age who do not currently smoke, what do you think about your chances of being **diagnosed** with lung cancer during your lifetime? Are you:	1 at much less risk<br>2 at less risk<br>3 at the same risk<br>4 at higher risk<br>5 at much higher risk<br>8 Don't know	◯	☐
**Passive Smoke Exposure** (PhenX code: 070300)			
D7.8 Were you exposed to smoke from other people's cigarettes or tobacco products during childhood at home?	1 yes 2 no 9 Don't know<br>If yes, for ______hours per day?<br>For how many years____	◯	☐
D7.9 Were you exposed to smoke from other people's cigarettes or tobacco products during your young- and middle-ages at home?	1 yes 2 no 9 Don't know<br>If yes, for ______hours per day?<br>For how many years____	◯	☐
D7.10 Were you exposed to smoke from other people's cigarettes or tobacco products in social settings such as restaurants or friends' homes?	1 yes 2 no 9 Don't know<br>If yes, for ______hours per day?<br>For how many years____	◯	☐
D8.1 Do you drink alcohol at the present time?	1 yes 2 no. *If "no", skip to 8.7*	◯	☐
D8.2 Did you drink alcohol in the past?	1 yes 2 no	◯	☐
If the answers of D8.1 is 'no', please skip to D9.1			
D8.3 How old were you when you began to drink alcohol?	age _______	◯	☐☐☐
D8.4 How old were you when stopped drinking alcohol if you don't drink alcohol at present?	age _______<br>*If still smoking now, please code 999;If don't remember when stopped, please code 888.*	◯	☐☐☐
D8.5 If you drink alcohol at the present time, what kind of alcohol do you drink?	1 very strong liquor (≥38%)<br>2 not very strong liquor (<38%)<br>3 wine 4 rice wine 5 beer 6 others	◯	☐
D8.6 If you drink alcohol at the present time, how much alcohol per day on average do you drink?	_______liang	◯	☐☐
**Alcohol - Lifetime Abuse and Dependence** (PhenX code#: 030501)			
D8.7 When you stop, cut down, or go without drinking, do you feel anxious for most of the day for 2 days or longer?	1 yes 2 no	◯	☐
D9.1 Do you do exercises regularly at present?	1 yes 2 no	◯	☐
D9.2 Did you do exercises regularly in the past?	1 yes 2 no	◯	☐

If the answers of D9.1 and D9.2 are both 'no', please skip to D10.1			
D9.3 How old were you when you began to do exercises?	age _______	○	□□□
D9.4 How old were you when you stopped doing exercises if you don't do exercises at present?	age _______<br>*If still doing exercise now, please code 999;*<br>*If don't remember when stopped, Please code 888.*	○	□□□
D10.1 Have you done physical labor regularly?	1 yes 2 no---*skip to D11*	○	□□□
D10.2 If yes, from which age	from age _______	○	□□□
D10.3 to which age?	to age _______	○	□□□

D11 Do you now perform the following activities regularly? (please choose one from frequency on the right)	1 almost everyday 2 not every day, but at least once a week 3 not every week, but at least once a month 4 not every month, but sometimes 5 never		
D11.1 Housework (cooking, taking care of kids)	1 2 3 4 5	○	□
D11.2 Personal outdoor activities	1 2 3 4 5	○	□
D11.3 Garden work	1 2 3 4 5	○	□
D11.4 Read newspapers/books	1 2 3 4 5	○	□
D11.5 Raise domestic animals	1 2 3 4 5	○	□
D11.6 Play cards and/or mah-jong	1 2 3 4 5	○	□
D11.7 Watch TV and/or listen to radio	1 2 3 4 5	○	□
D11.8 Social activities (organized)	1 2 3 4 5	○	□
D12 How many tours beyond home city/county have you made in the past two years?	_______ times *(if you have not made the tour, please code 00)*	○	□□

**E. ACTIVITIES OF DAILY LIVING (ADL) AND INSTRUMENTAL ACTIVITIES OF DAILY LIVING (IADL)**<br>For each area of functioning listed below, check the description that applies. (The word 'assistance' means supervision, direction, or personal assistance.)			Code
E0. For at least the last 6 months have you been limited in activities people usually do, because of a health problem? (If limited specify whether strongly limited or limited)	1. Yes, strongly limited<br>2. Yes, limited<br>3. Not limited	○	□

E1 **Bathing** – either sponge bath, tub bath, shower or washing the body	1 receives no assistance (gets in and out of tub alone if tub is usual means of bathing)---*skip to E2*<br>2 receives assistance in bathing only for part of the body (such as back or a leg)<br>3 receives assistance in bathing more than one part of the body (or doesn't bathe)	○	☐
E1.0 If receiving assistance, for how long?	_________ days	○	☐ ☐☐☐
E2 **Dressing** – gets clothes from closets and drawers – including underwear, outer garments and fasteners (including suspenders, if worn)	1 gets clothes and gets completely dressed without assistance--- *skip to E3*<br>2 gets clothes and gets dressed without assistance except for tying shoes<br>3 receives assistance in getting clothes or in getting dressed, or stays partly or completely undressed	○	☐
E2.0 If receiving assistance, for how long?	_________ days	○	☐ ☐☐☐
E3 **Toilet** – going to the toilet; cleaning oneself afterwards	1 goes to the toilet, cleans self, and arranges clothes without assistance (may use object for support such as cane, walker, or wheelchair)---*skip to E4*<br>2 can partly manage on his/her won, and receives assistance in going to the toilet or in cleaning self or in arranging clothes afterwards or in use of night bedpan or commode<br>3 bedridden and needs complete assistance in use of night bedpan or commode in bed.	○	☐
E3.0 If receiving assistance, for how long?	_________ days	○	☐ ☐☐☐
E4 **Indoor Transfer**	1 gets in and out of bed as well as in and out of a chair without assistance (may use object for support such as cane or walker)---*skip to E5*<br>2 gets in and out of bed or chair with assistance<br>3 bedridden	○	☐
E4.0 If receiving assistance, for how long?	_________ days	○	☐ ☐☐☐

E5 **Continence**	1 has complete control of urination and bowel movement without assistance--- *skip to E6*<br>2 has occasional 'accidents'<br>3 supervision helps keep urine or bowel control; catheter is used or elder is incontinent	○	□
E5.0 If has occasional 'accidents' or needs supervision, for how long?	_________ days	○	□□□□
E6 **Eating**	1 feeds self without assistance--- *skip to E6.1*<br>2 feeds self, with some help<br>3 receives assistance in feeding or is fed partly or completely intravenously	○	□
E6.0 If receiving assistance, for how long?	_________ days	○	□□□□
If the respondent chooses all 1 for E1 to E6, skip to E6.6			
E6.1 Who is the primary caregiver when you need assistance in above bathing, dressing, toileting, indoor transferring, continence, and eating?	1 spouse<br>2 son<br>3 daughter-in-law<br>4 daughter<br>5 son-in-law<br>6 unmarried son and daughter<br>7 grandchild(ren)<br>8 relative(s)<br>9 friends and neighbors<br>10 social services<br>11 housekeeper<br>12 nobody (*skip to E6.6*)	○	□□
E6.2 What is your primary caregiver's attitude when she/he takes care of you?	1 willing to do<br>2 impatience<br>3 need respite care<br>4 unwilling to do<br>5 don't know	○	□
E6.3 How much is the total direct cost last week paid for these caregiving?	______ Yuan (*if more than 100,000, please code 99998*)	○	□□□□□
E6.4 Who mainly pay the above cost?	1 self 2 spouse<br>3 children & their spouses<br>4 grandchildren & their spouses<br>5 state/collective 6 others	○	□
E6.5 Do you think the helps that you received in above six tasks could meet your needs?	1 fully meet<br>2 so so<br>3 unmeet		
E6.6 How many persons among your children, grandchildren and their spouses helped you in above six tasks last week?	_____ persons (*If nobody, fill 0 and skip to E7*)	○	□

E6.7 How many hours in total did your children, grandchildren and their spouses help you last week?	________ hours	◯	☐
E7 Can you visit your neighbors by yourself?	1 yes, independently<br>2 yes, but need some help<br>3 no, can't	◯	☐
E8 Can you go shopping by yourself?	1 yes, independently<br>2 yes, but need some help<br>3 no, can't	◯	☐
E9 Can you cook a meal by yourself whenever necessary?	1 yes, independently<br>2 yes, but need some help<br>3 no, can't	◯	☐
E10 Can you wash clothing by yourself whenever necessary?	1 yes, independently<br>2 yes, but need some help<br>3 no, can't	◯	☐
E11 Can you walk continuously for 1 kilometer at a time by yourself?	1 yes, independently<br>2 yes, but need some help<br>3 no, can't	◯	☐
E12 Can you lift a weight of 5kg, such as a heavy bag of groceries?	1 yes, independently<br>2 yes, but need some help<br>3 no, can't	◯	☐
E13 Can you continuously crouch and stand up three times?	1 yes, independently<br>2 yes, but need some help<br>3 no, can't	◯	☐
E14 Can you take public transportation by yourself?	1 yes, independently<br>2 yes, but need some help<br>3 no, can't	◯	☐

F. PERSONAL BACKGROUND			Code
F1 How many years did you attend school?	_______ *(if never, please code 00)*	◯	☐☐
F2 What was your primary occupation before age 60?	0 professional or technical personnel/doctors/teachers<br>1 governmental, institutional or managerial personnel<br>2 staff/service worker/industrial worker<br>3 self-employer<br>4 agriculture, forestry, animal husbandry, fishery<br>5 housewife<br>6 military personnel<br>7 unemployed<br>8 other, please specify:_____________	◯	☐
F2.1 Do you have a pension for retirement?	1 retired (worker) 2 retired (cadre) 3 no ---*skip to F3.1*	◯	☐
F2.1.1 Are you retired now?	1 retired (worker) 2 retired (cadre) 3 no ---*skip to F3.1*	◯	☐

F2.2 In which year did you retire if you have already retired?	_________ year	○	□□□□
F2. 2.2b What is your monthly pension?	___________ Yuan	○	□□□□□
F2.3 If you have retired, are you still engaged in paid jobs now?	1 yes 2 no	○	□
If having a pension for retirement, please skip to F3.1			
F2.4 Do you participated in public old age insurance?	1 yes 2 no---*skip to F2.7*	○	□
F2.5 What is the annual payment if participate in public old age insurance program?<br>--paid by individual:<br>--subsidy from collective or government:	______Yuan<br>______ Yuan	○	□□□□□<br>□□□□□
F2.5.b When did you initially participate in public old age insurance program?	______Year<br>______ Month	○	□□□□<br>□□
F2.6 What is your monthly pension from old age insurance at present?	_____Yuan---*skip to F3.1*	○	□□□□□
F2.7 What's the reason that you did not participate in public old age insurance program?	1 not worth<br>2 not necessary<br>3 cannot afford to the payment<br>8 don't know	○	□
F3.1 What is your primary means of financial support?	1 retirement wages<br>2 spouse 3 child(ren)<br>4 grandchild(ren) 5 relative(s)<br>6 local government or community<br>7 work<br>8 other, please specify:______________	○	□
F3.2 What is your other means of financial support? (multiple choices but limit to 5 choices)	1 retirement wages<br>2 spouse 3 child(ren)<br>4 grandchild(ren) 5 relative(s)<br>6 local government or community<br>7 work<br>8 other, please specify:______________<br>9 no other means	○	□□□□□
F3.3 Does all of your financial support sufficiently pay your daily costs?	1 yes 2 no	○	□
F3.4.0 Will you please tell me your status of decision making on financial spending in your household?	1 Make decisions on almost all spending in my household<br>2 Make decisions on my own spending and a small amount of other spending in my household<br>3 Make decisions only on my own spending	○	□

	4 Can't make decisions on any spending<br>5 Don't know		
F3.4 How do you rate your economic status compared with others in your local area?	1 very rich 2 rich 3 so so<br>4 poor 5 very poor 8 didn't answer	○	□
F3.5 What was the income per capita of your household last year?	________ Yuan (*if more than 100,000, please code 99998*)	○	□□□□□
F4.1 Current marital status:	1 married and living with spouse<br>2 married but not living with spouse<br>3 divorced<br>4 widowed<br>5 never married---*skip to F5*	○	□
F4.1a. Did you experience the following marital status change since you were interviewed last time for CLHLS study in 2011 (or 2012)?			
--Spouse passed-away (died)	1. Yes; 2. No		□
--Divorced	1. Yes; 2. No;<br>If yes, divorced in __month __year		□<br>□□, □□□□
--Remarried	1. Yes; 2. No;<br>If yes, remarried in __month __year		□□, □□□□
F4.1b. Do you have cohabited partner but not formally married at present?	1. Yes; 2. No ---*skip to F5*<br>If yes, cohabited in __month __year		□<br>□□, □□□□
F4.2 How many times have you been married?	__________, *if answer 0-- skip to F5*	○	□□

F4.3 Please tell me your marriage history		your age at this marriage	status of this marriage	age at marriage dissolution	good relationship?		age at this marriage	status	age at marriage dissolution	relationship
('age at marriage dissolution' to be answered only by divorced or widowed people)<br>*(If number of marriage is more than 4 times, please fill the last marriage in the cells of the 4th marriage)*<br>*(If the elder can't tell the exact age, the interviewer should help her/him recall an approximate one.)*	1st marriage		1 married<br>2 divorced<br>3 widowed		1 good<br>2 so so<br>3 bad	○	□□□	□	□□□	□
	2nd marriage		1 married<br>2 divorced<br>3 widowed		1 good<br>2 so so<br>3 bad	○	□□□	□	□□□	□
	3rd marriage		1 married<br>2 divorced<br>3 widowed		1 good<br>2 so so<br>3 bad	○	□□□	□	□□□	□
	4th marriage		1 married<br>2 divorced<br>3 widowed		1 good<br>2 so so<br>3 bad	○	□□□	□	□□□	□

F4.4 How many years did your last spouse attend school?	________<br>*If she/he never attended school, please '00')*	○	□□
F4.5 What was your last spouse's main occupation before age 60?	0 professional or technical personnel/doctors/teachers<br>1 governmental, institutional or managerial personnel<br>2 staff/service worker/industrial worker<br>3 self-employer<br>4 agriculture, forestry, animal husbandry, fishery<br>5 housewife<br>6 military personnel<br>7 unemployed<br>8 other, please specify:_____________	○	□
F4.6 Does your spouse have a paid job at present?	1 Yes, full time<br>2 Yes, part time 3 No	○	□
F4.7 How do you rate your spouse's health at present?	1 very good 2 good<br>3 so so 4 bad 5 very bad		□
F5 When you are sick, who usually takes care of you?	1 spouse<br>2 son<br>3 daughter-in-law<br>4 daughter<br>5 son-in-law<br>6 son and daughter<br>7 grandchildren and their spouses<br>8 other family members<br>9 friends<br>10 social services<br>11 live-in caregiver<br>12 nobody	○	□□
F6.1 Can you get adequate medical service when you are sick?	1 yes *(skip to F6.2)*<br>2 no	○	□
F6.1.0 What's the primary reason that you didn't go to the hospital when it was necessary?	1 no money to pay for expenses<br>2 far away<br>3 inconvenient in movement<br>4 nobody with whom to go<br>5 didn't want to go<br>6 other_____	○	□
F6.2 Could you get adequate medical service when you were sick at around age 60?	1 yes 2 no 8 didn't answer	○	□
F6.3 Could you get adequate medical service when you were sick in childhood?	1 yes 2 no 8 didn't answer	○	□

F6.4 Do you have following social security and commercialized insurances at present?<br><br>*If yes, fill in '1'; if no, fill in '0'.*	1 retirement pension<br>2 public old-age insurance<br>3 commercialized old age insurance<br>4 public free medical services<br>5 medical insurance for urban workers<br>6 collective medical insurance for urban residents<br>7 the new rural cooperative medical insurance<br>8 commercial medical insurance<br>9 other (please specify)_________	○	□<br>□<br>□<br>□<br>□<br>□<br>□<br>□<br>□
F6.5.1 How much did you spend on outpatient costs last year?	___________ Yuan (*if more than 100,000, please code 99998*)<br>Of which paid by family (self, spouse, children, etc.)<br>_______ Yuan	○	□□□□□<br>□□□□□
F6.5.1.a How much did you spend on inpatient costs last year?	___________ Yuan (*if more than 100,000, please code 99998*)<br>Of which paid by family (self, spouse, children, etc.)<br>_______ Yuan	○	□□□□□<br>□□□□□
F6.5.2 Who mainly pays these costs?	1 medical insurance for urban workers<br>2 **collective** medical insurance for urban residents<br>3 **the** new rural cooperative medical insurance<br>4 commercial medical insurance<br>5 self<br>6 spouse<br>7 children/grandchildren<br>8 no money to pay<br>9 others	○	□
F6.5.2.a How far from your home to the nearest hospital?	_______ kilometres		
F6.5.2.b Do you have regular phycial examination once every year?	1 yes 2 no		
F6.6 Did you frequently go to bed hungry as a child?	1 yes 2 no 8 didn't answer	○	□
F7.1 Is your mother alive?	1 yes 2 no---(*skip to F7.2.2*)<br>*(If the elder can't tell the exact age, the interviewer should help her/him recall an approximate one)*	○	□
F7.2.1 If so, how old is she?	_______ age --- (*skip to F8.1)*	○	□□□
F7.2.2 If not, how old was she when she died?	______ age (interviewers should help respondent to recall the age, see the survey manual)	○	□□□

F7.3 If she is dead, how old were you when she died?	_______ age<br>*(If the elder can't tell the exact age, the interviewer should help her/him recall an approximate one)*	○	□□□
F7.4 How many years did your mother attend school?	________ years<br>*(If she never attended school, fill '00'.)*	○	□□
F8.1 Is your father alive?	1 yes 2 no ---(*skip to F8.2.2)*	○	□
F8.2.1 If so, how old is he?	_______ age --- (*skip to F8.4)*<br>*(If the elder can't tell the exact age, the interviewer should help her/him recall an approximate one)*	○	□□□
F8.2.2 If not, how old was he when he died?	_______ age	○	□□□
F8.3 If he is dead, how old were you when he died?	_______ age<br>*(If the elder can't tell the exact age, the interviewer should help her/him recall an approximate one)*	○	□□□
F8.4 The main occupation of your father before age 60	0 professional or technical personnel/doctors/teachers<br>1 governmental, institutional or managerial personnel<br>2 staff/service worker/industrial worker<br>3 self-employer<br>4 agriculture, forestry, animal husbandry, fishery<br>5 housewife<br>6 military personnel<br>7 unemployed<br>8 other, please specify:_____________	○	□
F8.5 What was your father's occupation when you were a child?	0 professional or technical personnel/doctors/teachers<br>1 governmental, institutional or managerial personnel<br>2 staff/service worker/industrial worker<br>3 self-employer<br>4 agriculture, forestry, animal husbandry, fishery<br>5 housewife<br>6 military personnel<br>7 unemployed<br>8 other, please specify:_____________	○	□

F8.6 How many years of schooling did your father receive?	_______ (If he never attended school, fill '00'.)	○	□□
F9.1 What is your birth order among all of your biological siblings?	______ (If you have no sibling, fill '01' and skip to F10.)	○	□□

F9.2 Please tell me about your biological brothers and sisters who live elsewhere or have died, by birth order.

sex: 1 male 2 female

living or not: 1 yes 2 no

age: *If alive, fill in the age at present. If dead, fill in the age at death.*

frequent visits? 1 yes 2 no

residence:
1 in the same village/neighborhood
2 in the same township/district
3 in the same county/city
4 in a county/city nearby
5 elsewhere
8 unknown

(If alive, fill in the place where she/he lives at present. If dead, fill in the place where she/he lived before her/his death.)
(If the elder can't remember the exact age of a sibling, the interviewer should help her/him recall an approximate one)

birth order	sex	alive or not	age	frequent visits?	residence		birth order	sex	alive or not	age	visits?	residence
1						○	□□	□	□	□□□	□	□
2						○	□□	□	□	□□□	□	□
3						○	□□	□	□	□□□	□	□
4						○	□□	□	□	□□□	□	□
5						○	□□	□	□	□□□	□	□
6						○	□□	□	□	□□□	□	□
7						○	□□	□	□	□□□	□	□
8						○	□□	□	□	□□□	□	□
9						○	□□	□	□	□□□	□	□
10						○	□□	□	□	□□□	□	□
11						○	□□	□	□	□□□	□	□

name	Address, if alive and aged 80 or over

F10 How many children, including those who have died, do you have?	_______; Among them, ___ boy(s)	○	□□, □□
F10.1 Your age when gave the first birth	_______	○	□□
F10.2 Your age when gave the last birth	_______	○	□□

F10.3 Please tell me about all your children who live with you, live elsewhere or have died, by birth order.

Biological child: 1 yes 2 no

name	biological?	sex	alive?	age at present	frequent visits?	residence		biological?	sex	alive?	age at present	frequent visits?	residence
							○	□	□	□	□□□	□	□

**sex:**<br>1 male 2 female<br>**alive or not:**<br>1 alive 2 dead 3 unknown<br>**age at present:**<br>*If alive, fill in the age at present. If dead, how old would she/he be today?*<br>**frequent visits?**<br>1 yes 2 no<br>**residence:**<br>0 co-residence with the interviewee<br>1 in the same village/neighborhood<br>2 in the same township/district<br>3 in the same county/city<br>4 in the county/city nearby<br>5 elsewhere<br>8 unknown<br>*(If alive, fill in the place where she/he lives at present. If dead, fill in the place where she/he lived before her/his death.)*<br>*(If the elder can't remember the exact age of children, the interviewer should help her/him recall an approximate one)*								○	□	□	□	□□□	□	□
								○	□	□	□	□□□	□	□
								○	□	□	□	□□□	□	□
								○	□	□	□	□□□	□	□
								○	□	□	□	□□□	□	□
								○	□	□	□	□□□	□	□
								○	□	□	□	□□□	□	□
								○	□	□	□	□□□	□	□
								○	□	□	□	□□□	□	□
								○	□	□	□	□□□	□	□
								○	□	□	□	□□□	□	□
								○	□	□	□	□□□	□	□
								○	□	□	□	□□□	□	□

F10.4 If your eldest child has died, how old would s/he be at present if s/he were still alive?	______ *(skip to F10.5 if not applicable)*	○	□□□
F10.5 If your youngest child has died, how old would s/he be at present if s/he were still alive?	______ *(skip to F11.1 if not applicable)*	○	□□□
F11.1 To whom do you usually talk most frequently in daily life?<br>(Choose three)	0 spouse 1 son 2 daughter<br>3 daughter-in-law 4 son-in-law<br>5 grandchildren and their spouses<br>6 other relatives<br>7 friends/neighbors<br>8 social workers<br>9 housekeeper<br>10 nobody	○	First Second Third<br>□□ □□ □□
F11.2 To whom do you talk first when you need to tell something of your thoughts?	0 spouse 1 son 2 daughter<br>3 daughter-in-law 4 son-in-law<br>5 grandchildren and their spouses<br>6 other relatives<br>7 friends/neighbors<br>8 social workers<br>9 housekeeper<br>10 nobody	○	First Second Third<br>□□ □□ □□

F11.3 Who do you ask first for help when you have problems/difficulties?	0 spouse 1 son 2 daughter<br>3 daughter-in-law 4 son-in-law<br>5 grandchildren and their spouses<br>6 other relatives<br>7 friends/neighbors<br>8 social workers<br>9 housekeeper<br>10 nobody	○	First ☐☐ Second ☐☐ Third ☐☐
F12 How much money (including cash and value of materials) did you get last year from your children and their spouses both living and not living with you? (*if more than 100,000, please code 99998*)	sons and their spouses ________ Yuan / daughters and their spouses ______ Yuan / grandchildren _______ Yuan	○	sons and their spouses ☐☐☐☐☐ / daughters and their spouses ☐☐☐☐☐ / grand children ☐☐☐☐☐
F13 How much money (including cash and value of materials) did you give last year to your children and their spouses both living and not living with you? (*if more than 100,000, please code 99998*)	sons and their spouses ________ Yuan / daughters and their spouses ______ Yuan / grandchildren _______ Yuan	○	sons and their spouses ☐☐☐☐☐ / daughters and their spouses ☐☐☐☐☐ / grand children ☐☐☐☐☐
F14. What kind of social services are available in your community?<br>(1 Yes 2 No)	1 personal daily care services<br>2 home visits<br>3 psychological consulting<br>4 daily shopping<br>5 social and recreation activities<br>6 human rights consulting services<br>7 health education<br>8 neighboring relations<br>9 others(please specify)_________	○	☐<br>☐<br>☐<br>☐<br>☐<br>☐<br>☐<br>☐<br>☐
F15. What kind of social services do you expect to be provided by your community?<br>(1 Yes 2 No)	1 personal daily care services<br>2 home visits<br>3 psychological consulting<br>4 daily shopping<br>5 social and recreation activities<br>6 human rights consulting and services<br>7 health education<br>8 neighboring relations<br>9 others(please specify)_________	○	☐<br>☐<br>☐<br>☐<br>☐<br>☐<br>☐<br>☐<br>☐
F16. Which living arrangement do you prefer?	1 living alone (or with spouse), no matter how far children live<br>2 1 living alone (or with spouse), but it is better that live nearby<br>3 coresidence with children<br>4 institutions (elderly center, elderly home, etc.)<br>5 do not know	○	☐

G. OBJECTIVE EXAMINATION AND ILLNESSES ATTENTION:			Code
G0.1 How about the quality of your sleep?	1 very good 2 good 3 so so<br>4 bad 5 very bad	○	□
G0.2 How many hours do you sleep normally?	_______ hours	○	□
G1 Can the interviewee see a break in the circle on the cardboard sheet when lit by a flashlight and distinguish where the break is located?	1 can see and distinguish<br>2 can see only<br>3 cannot see 4 blind		□
G2.1 How many natural teeth does the interviewee have? (excluding false teeth)	______	○	□□
G2.2 Does the interviewee have false teeth?	1 yes 2 no	○	□
**Oral Hygiene of Personal Care** (PhenX code：080900)			
G2.3 How often do you brush your teeth?	-1 Not applicable (No teeth)<br>0 Do not<br>1 Once per day<br>2 Twice per day<br>3. Once or trice per day<br>4 More than trice per day<br>5 Sporadically<br>6 Unknown	○	□
**Toothache and Orofacial Pain** (PhenX code：081500)			
G2.4 During the past 6 months, did you have a toothache more than once, when biting or chewing?	1 Yes<br>2 No— *If "No", Skip to G2.5*	○	□
G2.4.1 On a scale of 1–10, where 1 is mild and 10 is severe, how would you rate this pain at its worst?	_____	○	□
G2.5 During the past 6 months, did you have pain in the jaw joint or in front of the ear or across your face or cheek more than once?	1 Yes<br>2 No-- *Skip to G3*	○	□
G2.5.1 On a scale of 1–10, where 1 is mild and 10 is severe, how would you rate this pain at its worst?	_____	○	□
G3 Can the interviewee use chopsticks to eat?	1 yes 2 no		□
**Hand Dominance** (PhenX code: 020600)			
G4 Which hand do you normally use to eat?	1 right-hand<br>2 left-hand	○	□

G4a Which hand do you normally use to write?	1 right-hand<br>2 left-hand<br>3 Never write	○	☐
G4b Which hand do you normally use to clean your teeth?	1 right-hand<br>2 left-hand<br>3 Never clean teeth	○	☐
G5. Blood pressure<br>G5.1 Systolic<br>G5.2 Diastolic	________mm mercury<br>________mm mercury		☐☐☐<br>☐☐☐
G6 Rhythm of heart	1 regular 2 irregular		☐
G7 Heart rate	________beats/min		☐☐☐
G8 Upper extremities - can interviewee put<br>G8.1 Hand behind neck	1 right 2 left<br>3 both 4 neither		☐
G8.2 Hand behind lower back	1 right 2 left<br>3 both 4 neither		☐
G8.3 Raise arms upright	1 right 2 left<br>3 both 4 neither		☐
G9 Can the interviewee stand up from sitting in a chair?	1 yes, without using hands<br>2 yes, using hands<br>3 no		☐
G10.1 Weight	______ kg		☐☐☐
**Standing height** (PhenX code 020703) **or self-reported height** (PhenX code 020704)			
G10.2a Standing Height, measured directly; if this is done, i.e., the height can be measured directly, skip to G10.2c<br>G10.2b Self-reported height	________cm; 9 cannot measure<br>________cm		☐☐☐<br>☐☐☐
**Waist Circumference** (Phenx code: 021600)			
G10.2c Waist Circumference	________cm		☐☐☐

Follow the procedures below to obtain this measure

The waist circumference measurement should be taken on bare skin.

1）Mark the measurement site: Stand on the participant’s right side. Palpate the hip area to locate the right ilium of the pelvis. You may ask the participant to locate his/her ilium before palpation. With the cosmetic pencil, draw a horizontal line just above the uppermost lateral border of the right ilium. Cross this mark at the midaxillary line, which extends from the armpit down the side of the torso. Exhibit 1 shows the anatomical location of the abdominal waist at the ilium. Repeat the same process on the participant’s left side.

2)Take the measurement: Make sure the participant does not inhale while his/her waist circumference is being measured and that the tape is not twisted. Wrap the tape measure around the individual’s waist as you would a belt, making sure that the zero end of the measure is at the beginning of the circumference. Use a retractable, tension-controlled steel measuring tape. When measuring the waist, be sure to position the tape in a horizontal plane at the level of the measurement mark. A wall mirror is useful to view the tape to ensure the horizontal alignment of the tape. Another person positioned on the opposite side of the participant should check that the tape sits parallel to the floor and lies snug but does not compress the skin. If a mirror or other person is not available, check the horizontal alignment of the tape before taking the measurement. Always position the zero end of the tape below the section containing the measurement value. Exhibit 1 demonstrates the correct placement of the tape at the ilium. Take the measurement to the nearest 0.1 cm at the end of the participant’s normal expiration.

3) Remove the tape measure and record the result.

4）Repeat the measurement.

Note: Tools are available that include a retractable tape with an anchoring pin that fits into the handle. These tools also assist the participant to lightly cinch the tape. If the investigator uses these tools, the protocol should be altered slightly to comply with directions of the manufacturer. See protocol B for use of this tool when measuring a different waist circumference. Detailed videos illustrating this procedure can be found on the NHANES website at: http://www.cdc.gov/nchs/products/elec_prods/subject/video.htm

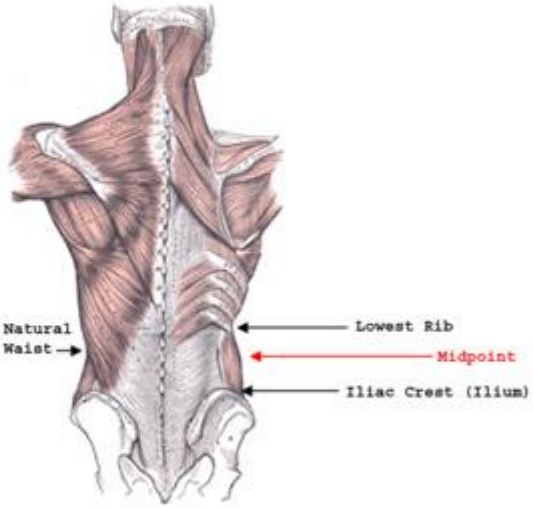

Exhibit 1:Location of Measurement Landmarks between the Lowest Rib and Iliac Crest (Ilium).

G10.2 Height, measured directly	________cm		□□□
If the height of the participant cannot be measured directly			
G10.2.1 Length from Acromion – processus to styloideus ulnae	________cm		□□□
G10.2.2 height from Right knee to the floor	________cm		□□□

How to indirectly measure height of the elderly (G10.2.1 and C10.2.2):
Please see the following figure for illustration on positions of Acrimion and processus styloideus ulnae.
The method for measuring distance from right knee to the floor is as follows:
Ask elder to take off right shoe;
(1) Ask elder to put the sole of his or her right foot onto the ground and to make his or her right calf and right thigh into a 90 degree angle.
(2) Put a plastic board or a thick paper on his or her right thigh levelly, and measure its height from ground with a ruler.

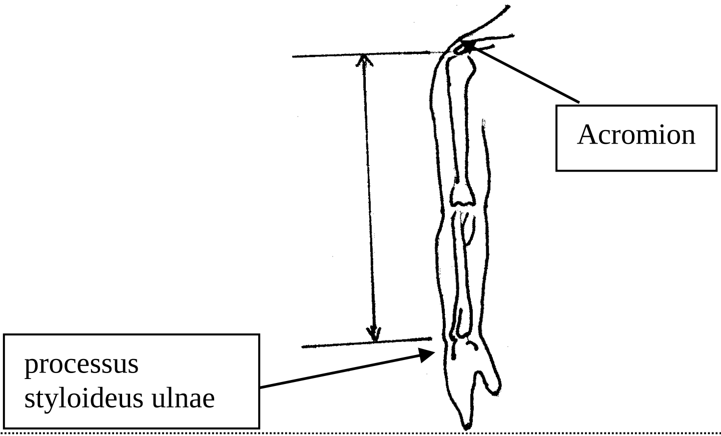

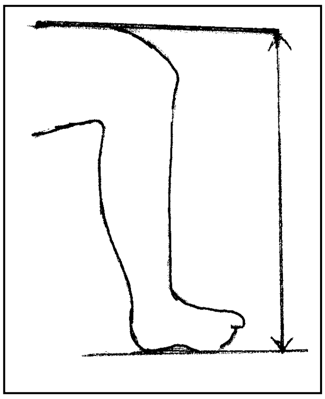

Hearing loss (PhenX code：201500)

G10.6 Do you have any difficulty with your hearing?	1 yes<br>2 no—*skip to G11*			□
G10.6.1 In which ear(s) do you have a hearing difficulty?	1 left 2 right 3 both			□
G10.6.2 At what age did you first notice a hearing difficulty?	1 I have had a hearing difficulty since I was born<br>2 My hearing difficulty developed during my childhood years (before the age of 15)<br>3 My hearing difficulty developed between the ages of 15 and 40<br>4 My hearing difficulty developed after the age of 40			□
G10.6.3 How quickly did your hearing difficulty develop?	1 Suddenly (over a few days)<br>2 Over a few months<br>3 Over several years			
G11 Was the interviewee able to pick up a book from the floor?	1 yes, standing<br>2 yes, sitting 3 no			□
G12 Was the interviewee able to turn around 360° without help?<br>*If yes, please count the number of steps required to finish the turn.*	______steps *(if no, fill in '88')*			□□

G13.0 Have you felt not well in the past two weeks?	1 yes 2 no	○	☐
G13 How many times have you suffered from a serious illness that required hospitalization or caused you to be bedridden at home in the past 2 years?	______ *(if no illnesses, fill in '00' and skip to G15; if permanently bedridden, fill in '88')*	○	☐☐
G14.1 Name of disease suffered and bedridden days at the first occurrence (Ref. Code in G15)	disease____________ days______		☐☐☐, ☐☐☐
G14.2 Name of disease suffered and bedridden days at the second occurrence (Ref. Code in G15)	disease____________ days______		☐☐☐, ☐☐☐
G14.3 Name of disease suffered and bedridden days at the third occurrence. If more than three occurrences, please ask for the last occurrence. (Ref. Code in G15)	disease____________ days______		☐☐☐, ☐☐☐

G15 Are you suffering from any of the following	yes or no<br>1 yes<br>2 no<br>3 don't know	Diagnosed by hospital?<br>1 yes 2 no	disability in daily life<br>1 rather serious<br>2 more or less<br>3 no	With disease or not?	Diagnosed by hospital or not?	Disability
01 Hypertension				☐	☐	☐
02 Diabetes				☐	☐	☐
03 Heart disease				☐	☐	☐
04 Stroke, cerebrovascular disease				☐	☐	☐
05 Bronchitis, emphysema, asthma, pneumonia				☐	☐	☐
06 Pulmonary tuberculosis				☐	☐	☐
07 Cataracts				☐	☐	☐
08 Glaucoma				☐	☐	☐
09 Cancer				☐	☐	☐
10 Prostate tumor				☐	☐	☐
11 Gastric or duodenal ulcer				☐	☐	☐
12 Parkinson's disease				☐	☐	☐
13 Bedsore				☐	☐	☐
14 Arthritis				☐	☐	☐
15 Dementia				☐	☐	☐

16 Epilepsy				☐	☐	☐
17 Cholecystitis, cholelith disease				☐	☐	☐
18 Blood disease				☐	☐	☐
19 Rheumatism or rheumatoid disease				☐	☐	☐
20 Chronic nephritis				☐	☐	☐
21 Galactophore disease				☐	☐	☐
22 Uterine tumor				☐	☐	☐
23 Hyperplasia of prostate				☐	☐	☐
24 Hepatitis				☐	☐	☐
25 Others, please specify:				☐	☐	☐

**H. QUESTIONS FOR INTERVIEWER**			Code
H1 Was the interviewee able to hear what you said?	1 yes, without hearing aid<br>2 yes, but needs hearing aid<br>3 partly, despite hearing aid<br>4 no		☐
H2.1 Was the interviewee able to participate in the physical check during the interview?	1 yes --- *skip to H3*<br>2 no 3 partially able to		☐
H2.2 If no or partially able, please give reason:	1 visually impaired, but can hear<br>2 hearing impaired, but can see<br>3 visually and hearing impaired<br>4 paralyzed<br>5 did not wish to participate<br>6 could not understand because of cognitive impairment<br>7 not able to participate at the moment because of some temporary illness such as a cold<br>8 other (please explain):<br>____________________		☐

H3 The interviewee was	1 surprisingly healthy (almost no obvious ailments)<br>2 relatively healthy (only minor ailments)<br>3 moderately ill (moderate degrees of major ailments or illnesses)<br>4 very ill (major ailments or diseases, bedridden, etc.)		□
H4 Date of birth printed on the individual ID Card<br><br>(**Note:** Those oldest old who were not issued individual ID Card according to local regulations, please fill date of birth printed on the household booklet.)	______year ____month ____day		□□□□ □□□□
H4.1 Was the date of birth printed on the Individual ID Card (or household booklet) the same as the self-reported age?	1 no<br>2 yes --- *skip to H5*<br>3 no self-reporting --- *skip to H5*<br>4 other (specify)___________		□
H4.2 If not, which one do you consider correct?	1 self-reported age<br>2 Individual ID Card or household booklet<br>3 not sure		□
H4.3 ID number printed on the individual ID Card (if ID card is not available for some oldest-old, please fill in "0")	____________________________		□□□□□□<br>□□□□□□<br>□□□□□□
H5 Please write the evidence for confirming the interviewee's age-reporting:<br><br>*(Regardless of whether or not there is self-reporting, you should confirm the age of the interviewee. If there is not enough space to write down your confirmation, please use the last page of this questionnaire).*	Age____<br><br>Evidence:<br><br>____________________________		□□□
H6 Have you checked whether you have failed to ask a question?	1 yes 2 no		□
H7 Did anyone help the interviewee to answer any question?	1 yes 2 no (*skip H7.1*)		□

H7.1 If yes, please check whether you have marked 'x' in the ❒ of the third column for those questions answered by people other than the interviewee. Please indicate mainly who helped to answer those questions.	1 spouse<br>2 child or spouse of child<br>3 grandchild or spouse of grandchild<br>4 great grandchild or spouse of great grandchild<br>5 sibling<br>6 parent or parent-in-law<br>7 caregiver or institutional staff<br>8 other, please specify_____		☐

**SPECIAL OBSERVATIONS**

**I. SPECIAL QUESTIONS (only applicable to those aged 105 or above)**		Code
Note to all persons who help to answer the questions listed below:<br>***According to Article 14 of Chapter 3 of the Law on Statistics, all information collected in this survey will be treated as strictly confidential. We will not tell anyone, including the elder him/herself, that you have helped to provide us information by answering the following questions, and your name will not be written down anywhere. The information collected here is purely for scientific research and nobody except qualified researchers will have access to this information. There will be NO connection between information collected here and the personal honor of the elder or any benefits she/he receives.***<br>***The elder's name: ______________ Sex:______ Code: ___________ Self-reported age:__________***		
S1 Information obtained from the elder's neighbors:<br>What is your opinion about this?<br>*(Present these possibilities and ask the respondent to choose one. The respondent should freely choose one based on the facts he or she knows.)*	1 I do not think the elder's age is correct.<br>2 I have doubts about the elder's age.<br>3 Perhaps the elder's age is correct, perhaps not – I do not know.<br>4 I suppose the elder's age is correct, but I do not know for sure.<br>5 I am absolutely sure the elder's age is correct.<br>*Whatever answer the respondent chooses please ask him or her to explain why:*<br>___________________________________	□
S2 Information obtained from the village leader or the neighborhood committee leader:<br>What is your opinion about this?<br>*(Present these possibilities and ask the respondent to choose one. The respondent should freely choose one based on the facts he or she knows.)*	1 I do not think the elder's age is correct.<br>2 I have doubts about the elder's age.<br>3 Perhaps the elder's age is correct, perhaps not – I do not know.<br>4 I suppose the elder's age is correct, but I do not know for sure.<br>5 I am absolutely sure the elder's age is correct.<br>*Whatever answer the respondent chooses please ask him or her to explain why:*<br>___________________________________	□
S3 Information obtained from the Aging Association officer:<br>What is your opinion about this?<br>*(Present these possibilities and ask the respondent to choose one. The respondent should freely choose one based on the facts he or she knows.)*	1 I do not think the elder's age is correct.<br>2 I have doubts about the elder's age.<br>3 Perhaps the elder's age is correct, perhaps not – I do not know.<br>4 I suppose the elder's age is correct, but I do not know for sure.<br>5 I am absolutely sure the elder's age is correct.<br>*Whatever answer the respondent chooses please ask him or her to explain why:*<br>___________________________________	□
S4 If there are genealogical records for the elder, please locate them and answer the following questions:		
S4.1 Birth date of the elder:	year____; month____; day___;	□□□□,□□,□□
S4.2 Date of first marriage of the elder:	year____; month____; day___;	□□□□,□□,□□

B Card 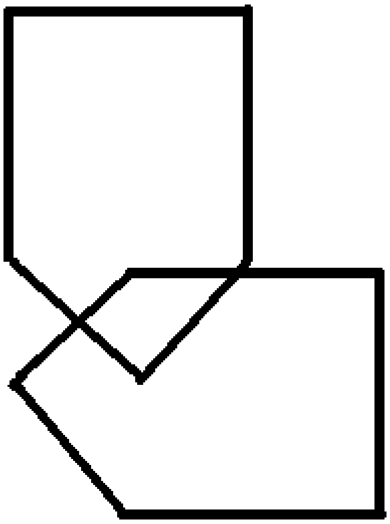
Please draw figure above here:
